# A Comparative Assessment of the Diagnosis of Swallowing Impairment and Gastroesophageal Reflux in Canines and Humans

**DOI:** 10.3389/fvets.2022.889331

**Published:** 2022-06-09

**Authors:** Tarini V. Ullal, Stanley L. Marks, Peter C. Belafsky, Jeffrey L. Conklin, John E. Pandolfino

**Affiliations:** ^1^Department of Medicine and Epidemiology, School of Veterinary Medicine, University of California, Davis, Davis, CA, United States; ^2^Department of Otolaryngology, Center for Voice and Swallowing, School of Medicine, University of California, Davis, Davis, CA, United States; ^3^The Vatche and Tamar Manoukian Division of Digestive Diseases, Department of Medicine, UCLA Robert G. Kardashian Center for Esophageal Health, David Geffen School of Medicine, University of California, Los Angeles, Los Angeles, CA, United States; ^4^Division of Gastroenterology and Hepatology, Department of Medicine, Northwestern Medicine, Feinberg School of Medicine, Northwestern University, Chicago, IL, United States

**Keywords:** esophageal anatomy, physiology, dysphagia, fluoroscopy, manometry, EndoFLIP®, gastroesophageal reflux

## Abstract

Swallowing impairment is a highly prevalent and clinically significant problem affecting people and dogs. There are myriad causes of swallowing impairment of which gastroesophageal reflux is the most common in both species. Similarities in anatomy and physiology between humans and canines results in analogous swallowing disorders including cricopharyngeus muscle achalasia, esophageal achalasia, hiatal herniation, and gastroesophageal reflux with secondary esophagitis and esophageal dysmotility. Accordingly, the diagnostic approach to human and canine patients with swallowing impairment is similar. Diagnostic procedures such as swallowing fluoroscopy, high-resolution manometry, pH/impedance monitoring, and endolumenal functional luminal imaging probe can be performed in both species; however, nasofacial conformation, increased esophageal length, and the difficulty of completing several of these procedures in awake dogs are inherent challenges that need to be considered. Human patients can convey their symptoms and respond to verbal cues, whereas veterinarians must rely on clinical histories narrated by pet owners followed by comprehensive physical examination and observation of the animal eating different food consistencies and drinking water. Dogs may also be unwilling to drink or eat in the hospital setting and may be resistant to physical restraint during diagnostic procedures. Despite the species differences and diagnostic challenges, dogs are a natural animal model for many oropharyngeal and esophageal disorders affecting people, which presents a tremendous opportunity for shared learnings. This manuscript reviews the comparative aspects of esophageal anatomy and physiology between humans and canines, summarizes the diagnostic assessment of swallowing impairment in both species, and discusses future considerations for collaborative medicine and translational research.

## Introduction

Difficulty swallowing is a prevalent problem in both people and dogs (1–3) that can cause malnutrition (4), dehydration (5), aspiration pneumonia (6, 7), and negatively impact overall quality of life (8). The exact prevalence of swallowing impairment in dogs is unknown, but at the University of California, Davis, nearly 1% of 105,000 dogs presenting to the Small Animal Clinic between 2003 and 2013 were evaluated for a swallowing abnormality. In humans, 1 in 6 adults in the US report symptoms of dysphagia (9) and contribute to nearly 600,000 outpatient visits yearly (1). Dysphagia is even more common in the elderly population with a prevalence of 15% (10). The prevalence of difficulty swallowing is more easily documented in human patients because they can report their symptoms of dysphagia. The term “dysphagia” denotes symptoms that canine patients cannot convey. Thus, although dysphagia is still conventionally used in veterinary medicine to describe swallowing impairment in dogs and cats, the authors have elected to use the term “swallowing impairment” over “dysphagia” in this manuscript to more accurately portray this important phenomenon.

Swallowing impairment can be categorized anatomically into oropharyngeal or esophageal disorders and further classified into structural, motility, or functional disorders (11). Functional disorders are unique to human patients because they can report their symptoms, including pain while swallowing (odynophagia) or a sensation of food sticking in the throat or chest. Extensive diagnostic testing excludes structural and motility disorders to diagnose a functional disorder (12).

Functional and motility disorders can involve not just the oropharynx and esophagus, but the entire gastrointestinal tract (13). For example, systemic scleroderma (14–16) and dysautonomia (17, 18) can cause esophageal and gastrointestinal dysmotility (14–18). Thus, a holistic assessment of the gastrointestinal tract is valuable when evaluating patients with swallowing impairment.

Evaluating gastrointestinal motility is specifically important in patients with gastroesophageal reflux disease (GERD). Delayed gastric emptying from an outflow obstruction (19), gastroparesis (20), or ileus (21) can exacerbate gastroesophageal reflux (GER) and esophageal dysmotility (20, 22, 23). Methods such as gastric emptying scintigraphy (24, 25), ultrasound (26, 27), or wireless motility capsules (28, 29) can be employed to assess gastrointestinal motility. However, an extensive review of gastrointestinal motility and its assessment is beyond the scope of this manuscript.

Prior to pursuing advanced diagnostic tests in patients with swallowing impairment, the clinician should obtain a thorough clinical history and patient examination to help distinguish the anatomic location and cause of the swallowing impairment. For example, dropping of food from the mouth in dogs is characteristic of oral dysfunction that may be secondary to poor dentition, glossal disease, or oral tumors. Pharyngeal and cricopharyngeus muscle impairment often cause gagging and retching within seconds of food or water consumption whereas regurgitation is more delayed with esophageal or gastroesophageal disorders (30). Humans may localize concerns to a specific area of discomfort. Examples include globus (31), which is a non-painful sensation of tightness in the pharynx; heartburn, a burning sensation in the retrosternal region; or dyspepsia, characterized by epigastric pain (32, 33). A history of a stroke or signs such as muscle atrophy, generalized weakness, tremors, or cognitive decline suggest neuromuscular pathology. Nevertheless, in light of many overlapping clinical signs and the breadth of possible differential diagnoses in both dogs and people, further diagnostic tests are usually needed to accurately localize and confirm the underlying etiology of the swallowing impairment.

There is a plethora of causes of swallowing impairment, but due to similarities in pharyngeal and esophageal anatomy and function between humans and canines (34–37), many of the same diseases occur. Some of the most common causes of swallowing dysfunction in both canines and humans include cricopharyngeus muscle achalasia (38, 39), esophageal achalasia (40, 41), sliding (Type I) hiatal herniation (42–44), GER (32, 43, 45) with secondary esophagitis (46–48), esophageal strictures (49), and esophageal dysmotility (50, 51). Thus, many diagnostic procedures utilized to assess human patients can be used in dogs. Examples include swallowing fluoroscopy (2, 52), high-resolution manometry (HRM) (53), pH/impedance monitoring (45, 54), and endolumenal functional luminal imaging probe (EndoFLIP) (40, 55–58). However, there are differences between the two species in patient conformation, neuromuscular anatomy, and compliance that impact performing and interpreting these tests in canine patients. Furthermore, there are disparities between the human and veterinary fields in research progress, funding, and equipment availability that curb the widespread use of these diagnostic modalities in veterinary medicine. For example, HRM and endoFLIP hardware and software currently cost $70,000 and $81,000, respectively, which can be cost-prohibitive for veterinary clinics to purchase. In addition, the HRM esophageal catheters are extremely fragile, have a limited number of usages, and cost $16,000 to replace.

This review article will highlight the comparative aspects of esophageal anatomy and physiology between humans and canines, summarize the procedures and applications of swallowing fluoroscopy, HRM, pH/impedance, and EndoFLIP to assess swallowing impairment in both species, explain the limitations and roadblocks to using these tests in canines, and discuss future directions and considerations for collaborative medicine and translational research.

## Comparative Esophageal Anatomy and Physiology Between Humans and Dogs

### Functions of the Esophagus

The esophagus is an essential neuromuscular tubular structure that functions to transport food or liquid from the pharynx to stomach. The upper (UES) and lower esophageal sphincters (LES) relax to allow ingesta into the esophagus and stomach, respectively. The UES and LES are otherwise tonically contracted to block laryngopharyngeal and gastroesophageal reflux, respectively, and prevent aspiration (34–37, 59). The UES also prevents entry of excess air into the digestive tract (60). The conservation of these esophageal and sphincter functions across species explains the similarities in anatomy and physiology between humans and canines.

### Anatomical and Physiological Similarities

**Figure 1:**
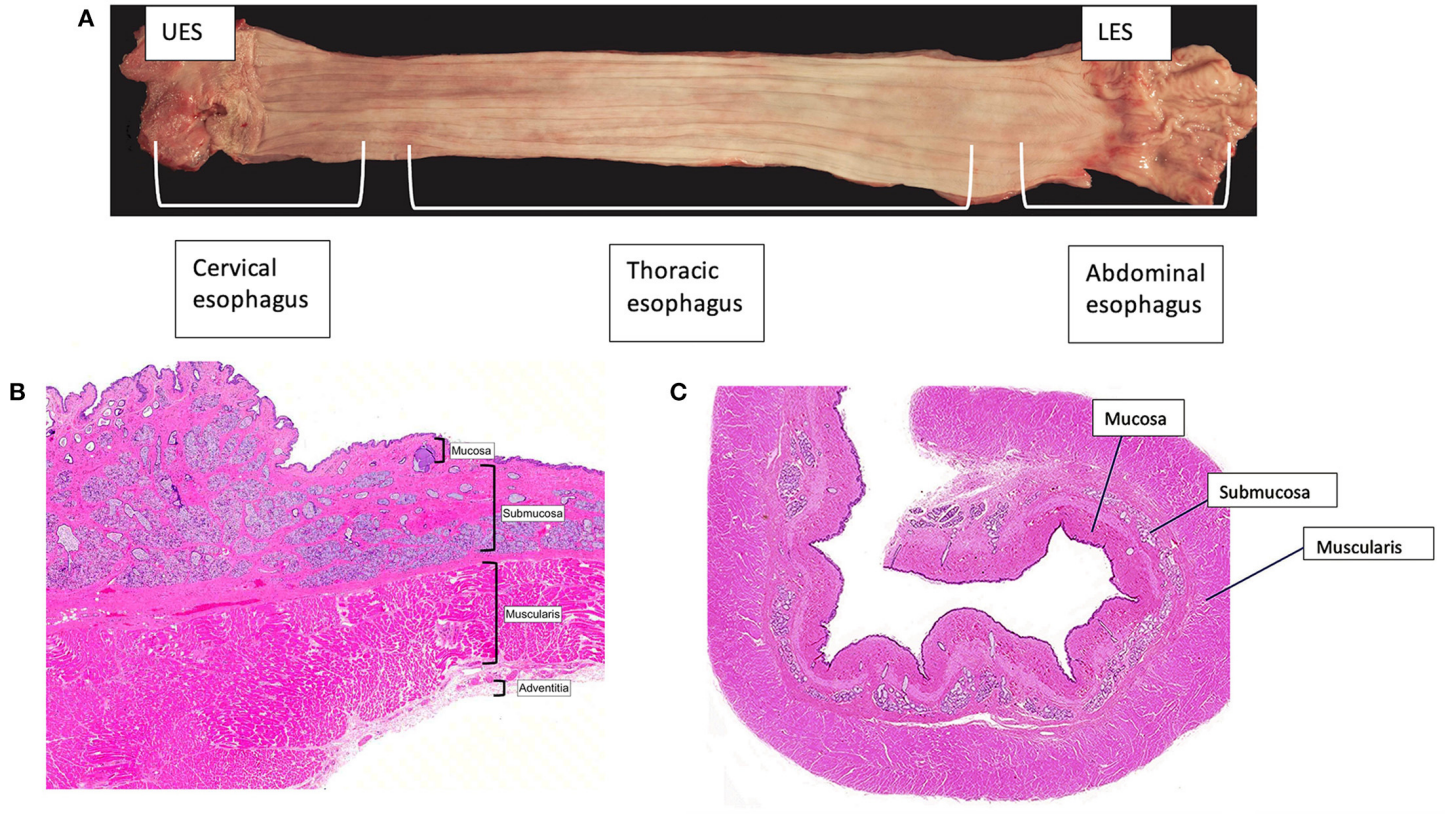
Esophageal anatomy. **(A)** The canine esophagus is shown with the proximal esophagus on the left and the distal on the right. The canine and human esophagus are composed of the upper esophageal sphincter, the esophageal body, which is segmented into the cervical, thoracic, and abdominal esophagus, and the lower esophageal sphincter. **(B,C)** A transverse image from the cervical portion of the canine esophagus **(B)** and the thoracic portion of the canine esophagus **(C)**. Images were obtained with light microscopy and stained with hematoxylin and eosin. The inner folded mucosal layer is surrounded by submucosa, muscularis, and the outermost external adventitia. Note the absence of a serosal layer in the esophagus. This makes the esophagus reliant upon the holding strength of the submucosa.

The basic anatomy of the human and canine esophagus is organized into two zones of high pressure at the UES and LES with an esophageal body between that is divided into cervical, thoracic, and abdominal components (Figure 1A). The wall of the esophagus consists of 4 separate tissue layers: mucosa, submucosa, muscularis, and the outermost adventitia (Figures 1B,C) (16, 17, 19). The mucosa is composed of stratified squamous epithelial cells linked together by tight junctions, desmosomes, claudins, occludins, and other fortifying proteins to create a protective barrier (61). Atop the epithelial surface rests a pre-epithelial layer of bicarbonate, mucin, and water that neutralizes swallowed or refluxed acidic contents. The components of the pre-epithelial layer are secreted by submucosal mucous glands. Other elements of the submucosa are blood vessels, nerves, and collagenous connective tissue (35, 37, 59).

**Figure 2:**
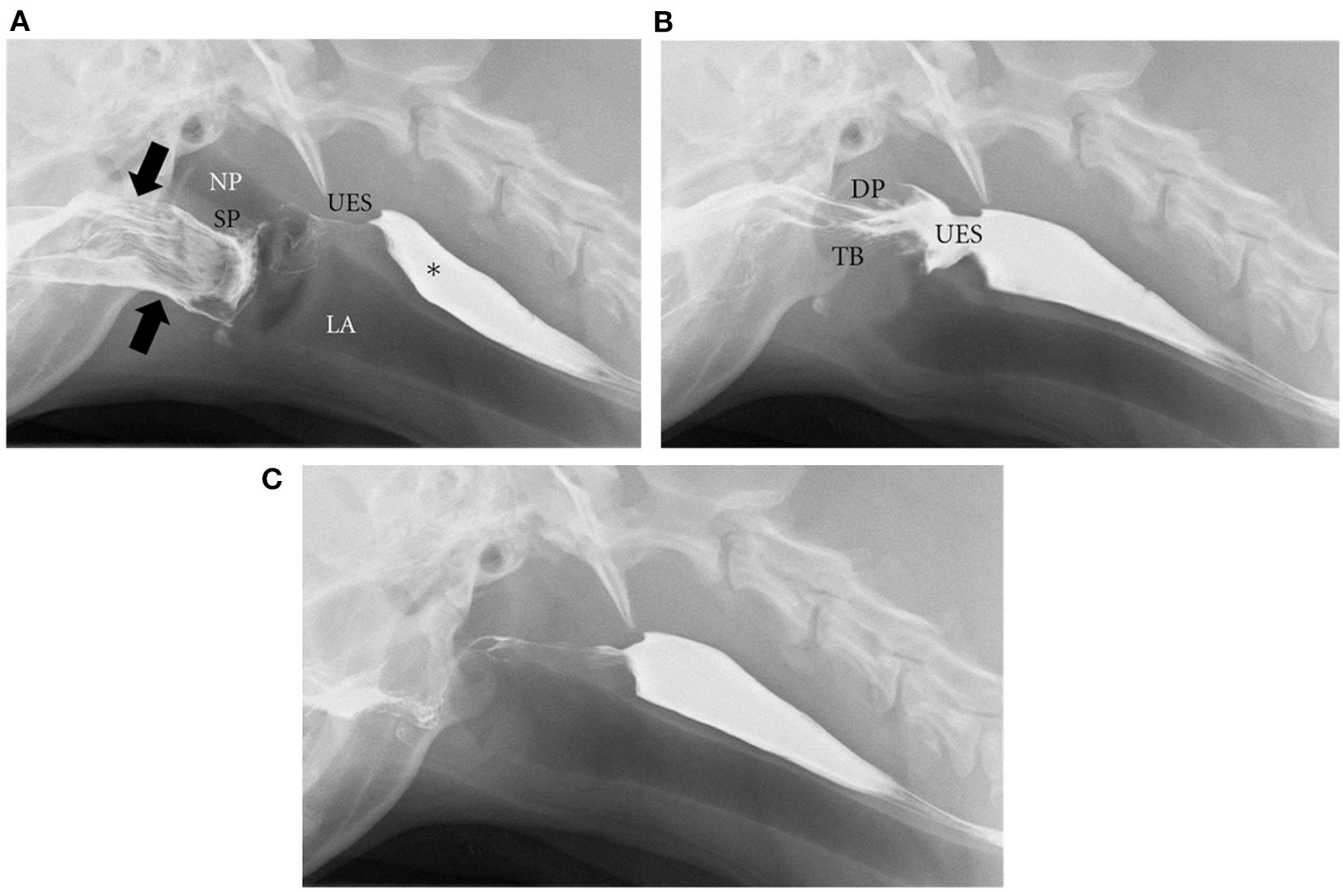
Phases of deglutition. Digital images from a videofluoroscopic swallow study in a healthy dog show the phases of deglutition. **(A)** Oral phase: Liquid barium contrast in the oral cavity (designated by black arrows). There is also remaining barium from a previous swallow in the cervical esophagus (*). At the start of the pharyngeal phase, the soft palate will rise to close the nasopharynx while the epiglottis closes the larynx to prevent nasopharyngeal reflux and laryngeal penetration, respectively. NP, nasopharynx; LA, larynx; SP, soft palate; UES, upper esophageal sphincter. **(B)** As a continuation of the pharyngeal phase, the pharyngeal muscles contract and the dorsal pharyngeal wall (DP) meets the tongue base (TB) while the cricopharyngeus muscle relaxes to open the upper esophageal sphincter (UES). Liquid barium contrast can then pass through the open UES into the proximal esophagus. **(C)** After the contrast reaches the esophagus, esophageal peristalsis (primary and secondary) can occur to move the bolus through the lower esophageal sphincter (LES) to the stomach. **(A–C)** were reprinted from International Scholarly Research Network Veterinary Science, Volume 2012, Pollard RE, Imaging evaluation of dogs and cats with dysphagia, Copyright 2012 Rachel E. Pollard. Reprinted with permission from Dr. Rachel E Pollard.

The integrity and coordination of these anatomical components enables normal deglutition in humans and canines. Deglutition (Figures 2A–C) (2) begins with the oral preparatory phase, which is voluntary and is associated with mastication and lubrication of the food bolus in preparation for swallowing. The oral phase consists of the muscular events responsible for movement of the bolus from the tongue to the pharynx, and is facilitated by the tongue, jaw, and hyoid muscle movements. The pharyngeal phase begins as the bolus reaches the tonsils, and is characterized by elevation of the soft palate to prevent the bolus from entering the nasopharynx, elevation and forward movement of the larynx and hyoid, retroflexion of the epiglottis and closure of the vocal folds to close the entrance into the larynx, synchronized contraction of the middle and inferior constrictor muscles of the pharynx, and relaxation of the cricopharyngeus muscle, which makes up much of the UES, to allow passage of the bolus into the esophagus. Respiration is briefly halted (apneic moment) during the pharyngeal phase. The esophageal phase follows during which peristaltic contractions drive contents down the esophageal body, across the esophagogastric junction (EGJ), and into the stomach (59, 62–64). Primary peristalsis is triggered in the swallowing center by activation of vagal lower motor neurons, which interact with neuromuscular elements of the esophageal wall. Peristaltic contractions then spearhead bolus transit. Secondary peristalsis, stimulated by mechanical distension of the esophagus and enhanced by chemosensory triggers (65), assists to clear ingested material not cleared by primary peristalsis (66). Multiple rapid swallows induce a period of latency called deglutitive inhibition that terminates with an accentuated peristaltic contraction. This physiologic pattern has been observed in both humans and dogs (53, 67). Transient lower esophageal sphincter relaxation (tLESR) is a vagally-mediated reflex that also occurs in both species. Gastric distension stimulates stretch receptors, which activate vagal sensory neurons that synapse on interneurons in the nucleus tractus solitarius of the brainstem. These interneurons then excite vagal motor neurons in the dorsal motor nucleus of the vagus, which travel in the vagus to stimulate myenteric neurons that innervate LES muscle. The myenteric neurons make nitric oxide, which causes LES relaxation. This is a major mechanism of GER and is the genesis of the gastric belch (68, 69).

### Similarities in Pathology

Given the resemblance in esophageal anatomy and physiology between humans and canines, it is logical that the two species share common pathologies. The most common anatomic locations involved in swallowing impairment are oropharyngeal and esophageal pathologies.

#### Oropharyngeal Swallowing Impairment

Oropharyngeal swallowing impairment can be the result of oral, palatal, pharyngeal or pharyngoesophageal pathology. In humans, oropharyngeal swallowing impairment is particularly common amongst geriatric patients (70), secondary to aging or associated neurologic conditions. Elderly human patients have reduced lingual propulsion and delayed swallow response (70). They also have a smaller UES diameter, which could be due to reduced UES compliance or webbing, weak pharyngeal drive, and decreased hyolaryngeal traction (71, 72). In addition to aging, several neurologic conditions such as stroke (73), Alzheimer's disease (74), Parkinson's disease (75), neuromuscular diseases, and dementia (76) can cause pharyngeal weakness, discoordination, and/or UES dysfunction in humans. Similarly, pharyngeal weakness in dogs mainly occurs secondary to neuromuscular disorders [myasthenia gravis (77), muscular dystrophy (78), polymyositis (79), and polyneuropathies (80)]. Pharyngeal weakness typically occurs in middle-aged to older dogs, and can cause delayed propulsion of the bolus to the UES, with subsequent asynchrony between pharyngeal contraction and relaxation of the UES (81).

#### Esophageal Swallowing Impairment

**Figure 3:**
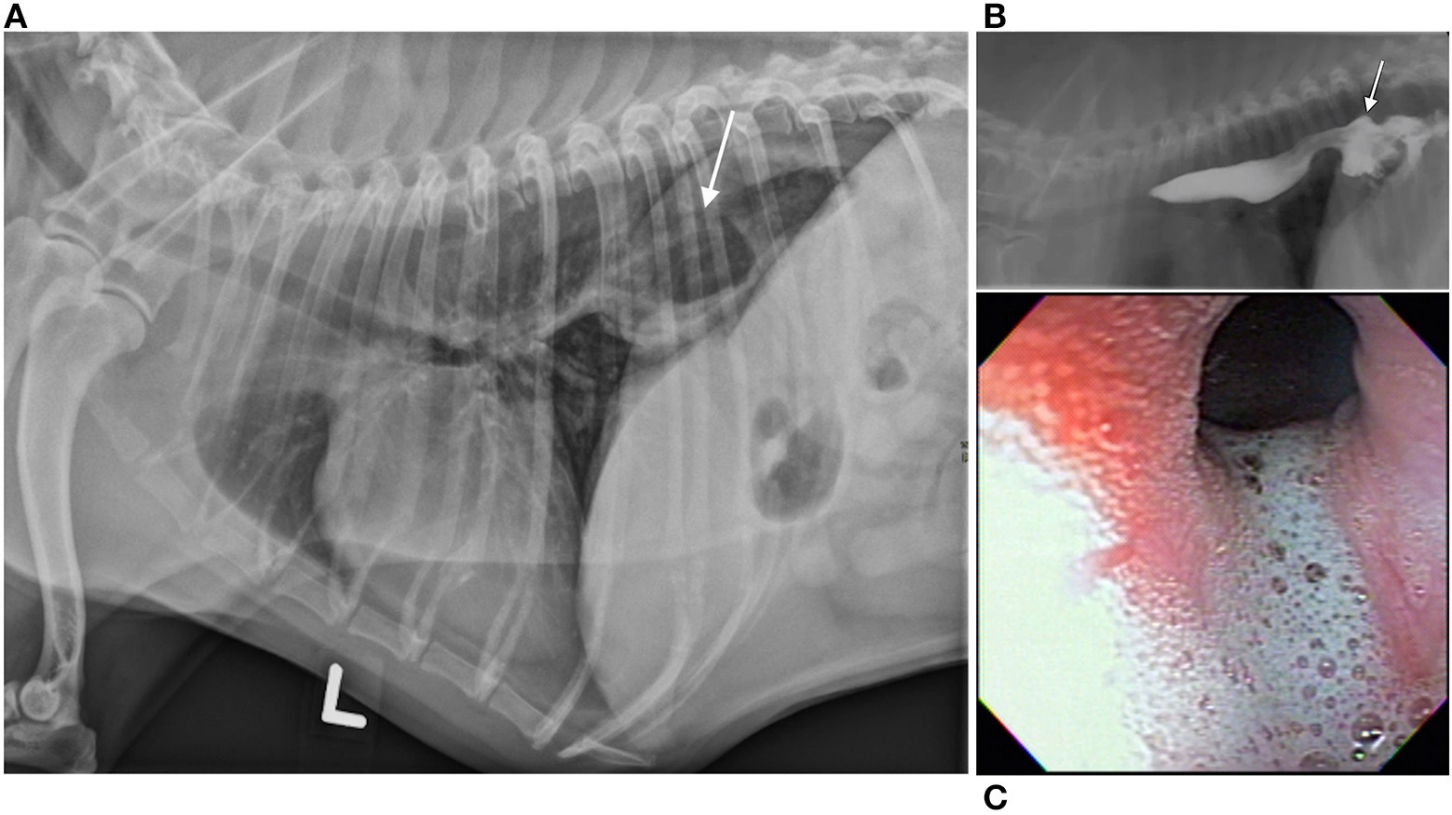
Hiatal herniation, gastroesophageal reflux, and esophagitis in a brachycephalic dog. Hiatal herniation in a brachycephalic dog. **(A)** Left lateral thoracic radiograph of a 5-year old brachycephalic Boston Terrier with a chronic history of regurgitation. The stomach is seen extending through the diaphragm into the craniodorsal thorax in this image (arrow). Although not pictured, the stomach returns to a normal position on subsequent views, which is suggestive of a sliding or type I hiatal hernia. **(B)** Contrast videofluoroscopic swallow study of the same patient. This image documents gastroesophageal reflux of barium contrast as a result of hiatal herniation (arrow points to hiatal herniation and stream of white contrast extending cranial is evidence of gastroesophageal reflux). **(C)** Endoscopic image from the same patient showing foamy gastroesophageal reflux cranial to the lower esophageal sphincter and secondary esophagitis [reddened hyperemic area in the upper left of the image (denoted with arrow)]. **(A–C)** Images were published in Textbook of veterinary internal medicine: diseases of the dog and the cat, Vol 2, 8th edition, Marks SL, Chapter 273: Diseases of the Pharynx and Esophagus, 8501–8576, Copyright 2017 by Elsevier, Inc, Reprinted with permission from Elsevier.

**Figure 4:**
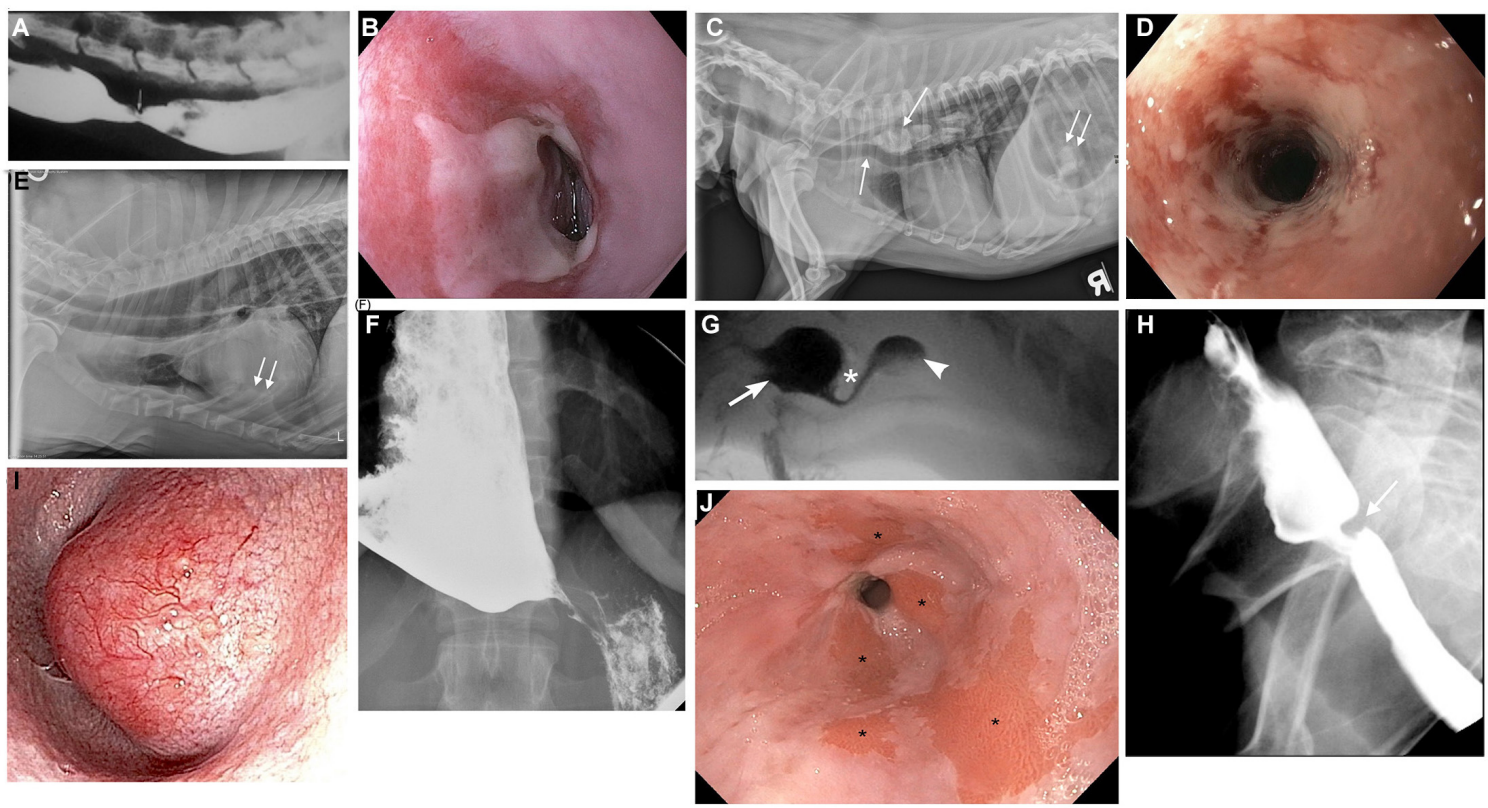
Canine and human esophageal disorders. **(A)** Contrast esophagram study performed in lateral recumbency in a 2-year old mixed breed dog documenting a focal esophageal stricture (arrow) secondary to severe gastroesophageal reflux. **(B)** Endoscopic image of a peptic stricture secondary to gastroesophageal reflux in a 58-year-old human patient. **(C)** Survey lateral thoracic radiograph of a 7-year old mixed breed dog with multiple fragments of a pork bone lodged in the thoracic esophagus (downward arrow). The mineral fragments are distending the esophagus and even ventrally deviate the trachea (upward arrow). There are also a few mineral fragments seen in the gas-dilated stomach (double arrow). **(D)** Severe ulcerative esophagitis and an esophageal stricture in a 48-year old human patient with a history of gastroesophageal reflux. **(E)** Right lateral survey thoracic radiograph of a 3-year old male Viszla with a 3-week history of regurgitation, ptyalism, and dysphonia. The esophagus is diffusely gas-distended (arrow) and there are ventral interstitial to alveolar infiltrates within the left cranial and right middle lung lobes (double arrows) consistent with aspiration pneumonia. The dog was diagnosed with focal myasthenia gravis and the megaesophagus resolved with pyridostigmine treatment of the myasthenia gravis. **(F)** Anterior-posterior contrast radiographic image of a 35-year old human patient with a sigmoid megaesophagus secondary to achalasia. The distal esophagus is distended with barium contrast, but the contrast column narrows into a classic bird's beak shape at the esophagogastric junction due to failed relaxation of the lower esophageal sphincter. **(G)** Videofluoroscopic still image from a 7-month-old spayed female miniature Dachshund with severe dysphagia secondary to cricopharyngeal achalasia. A hypertrophied cricopharyngeus muscle (cricopharyngeal bar) is seen (asterisk), which obstructs bolus passage of the barium liquid from the pharynx (arrow) into the proximal esophagus (arrowhead). The barium column seen below the asterisk is attenuated as it flows through the narrow opening of the upper esophageal sphincter (UES). **(H)** Videofluoroscopic still image from a 78-year-old human patient with a cricopharyngeal bar. A fibrotic cricopharyngeus muscle (cricopharyngeal bar) is seen (arrow) that obstructs bolus passage of barium liquid from the pharynx into the proximal esophagus. **(I)** Endoscopic image of a distal esophageal mass in a 13-year-old male West Highland White Terrier with a history of lip-smacking and regurgitation. The mass had a broad-based attachment to the esophageal mucosa on esophagoscopy, but was surgically resected with marginal excision to confirm a well-differentiated leiomyosarcoma. **(J)** Endoscopic image of a 62-year-old human patient with a history of chronic gastroesophageal reflux and subsequent Barrett's esophagus. The salmon-colored patches of mucosa (asterisks) in the distal esophagus are areas where squamous epithelium has converted to metaplastic columnar epithelium as a result of chronic esophageal mucosal injury. This patient is at an increased risk of developing esophageal cancer. **(A,C,E,G)** Images were published in Textbook of veterinary internal medicine: diseases of the dog and the cat, Vol 2, 8th edition, Marks SL, Chapter 273: Diseases of the Pharynx and Esophagus, 8501–8576, Copyright 2017 by Elsevier, Inc, Reprinted with permission from Elsevier.

Esophageal swallowing impairment in canines is mainly caused by gastroesophageal disease with consequent esophagitis, structural lesions, or motility disorders. In both dogs and people, gastroesophageal disease due to GER ± hiatal herniation is the most common etiology (42–45). Brachycephalic (short-muzzled) breeds (French bulldogs, English bulldogs, pugs, and Boston terriers, boxers, shih-tzus) are frequently affected (Figures 3A–C) (82). Due to their nasofacial conformation and unique respiratory anatomy, they often have an upper airway tract obstruction, brachycephalic obstructive airway syndrome (BOAS), which increases negative intrathoracic pressure and causes subsequent hiatal herniation and GER (42, 50, 83). GER in dogs also frequently occurs during anesthesia secondary to reduction of LES tone (84, 85), and can lead to reflux esophagitis, esophageal dysmotility, and esophageal strictures (86) identical to peptic strictures in humans (Figures 4A,B,D) (32, 82). Other structural lesions such as tumors or vascular ring anomalies (87) occur in both species although dogs are at greater risk for esophageal foreign bodies (88) (Figure 4C) (82). Motility disorders in canine patients are frequently found in association with megaesophagus, which can be a congenital or acquired disease in dogs (Figure 4E) (82). Approximately 50% of dogs with megaesophagus have an acquired and idiopathic form, but there are many secondary causes of megaesophagus associated with polyneuropathies, polymyopathies, junctionopathies (myasthenia gravis, botulism, tick paralysis, tetanus, organophosphate poisoning), myopathies (inflammatory myopathies, dermatomyositis), and polyneuropathies (polyradiculoneuritis, dysautonomia) (89) in dogs. In humans, a sigmoid megaesophagus can develop as an end result of late-stage achalasia (90) (Figure 4F). Similarly, megaesophagus secondary to LES achalasia has been described in dogs and at one academic institution's teaching hospital, comprised 60% (14/23) of the megaesophagus cases seen over a 2-year span (40, 41). Esophageal motility disorders in the absence of megaesophagus such as juvenile esophageal dysmotility (51) or dysmotility secondary to GER have been identified in dogs (3, 42) but esophageal motility disorders in dogs are poorly characterized compared to those in humans in light of the limited application of HRM in animals (91).

### Differences in Pathology and Anatomy

Despite the similarities, there are numerous differences in pathology and anatomy between the two species. Firstly, although cricopharyngeus muscle dysfunction can affect older dogs, it is far more commonly recognized in young puppies as a congenital anomaly in the form of cricopharyngeus muscle asynchrony (delayed UES opening) or achalasia (ineffective UES opening) (71, 72) (Figure 4G) (82). A hereditary cause for cricopharyngeus muscle dysfunction has been identified in Golden Retrievers, and results of complex segregation analysis suggest that a single recessive allele of large effect contributed to the expression of this disease in the breed (92). In addition, miniature dachshunds, Maltese, toy poodles, and spaniels are predisposed to the congenital development of cricopharyngeus muscle dysfunction (Table 1) (92–95).

**Table 1:** Breed associations of oropharyngeal and esophageal swallowing disorders in dogs.

**Classification**	**Disorder**	**Breed association**
Oropharyngeal	Masticatory muscle myositis	German shepherds Labrador retrievers, Doberman pinschers Golden retrievers (120) Cavalier King Charles spaniels (SLM—personal communication)
Oropharyngeal	Trigeminal neuropathy	Golden retriever (121)
Oropharyngeal	Cricopharyngeus muscle dysfunction (achalasia and asynchrony)	Miniature dachshund (93) Cocker spaniel (94) Springer spaniel (95) Cavalier King Charles spaniel (SLM—personal communication) Maltese (93) Toy poodle (SLM—personal communication) Golden retriever (92)
Oropharyngeal	Pharyngeal dysphagia and masticatory muscle atrophy	Hungarian vizslas (122)
Oropharyngeal/esophageal	Distal muscular dystrophy and sarcoglycan deficient muscular dystrophy	Bouvier des flandres (78) Golden retriever (123, 124) Labrador retriever (123, 125) Cavalier King Charles spaniel (126, 127) Miniature dachshund (128) Alaskan malamute (129) Lurcher (130) Rottweiler (127) Boston terrier (131, 132)
	Inflammatory polymyopathy	Boxer (133, 134) Newfoundland (134) Pembroke Welsh Corgi (135)
Esophageal	Congenital megaesophagus Secondary to congenital Myasthenia gravis*	Miniature Schnauzer Smooth fox Terrier* Newfoundland Parson Russell terrier* Samoyed Shar-pei Springer spaniel* (89)
Esophageal	Acquired idiopathic megaesophagus Acquired megaesophagus secondary to Myasthenia gravis	Irish setter Great dane German shepherd Labrador retriever Miniature schnauzer Newfoundland (7, 89) Akitas Scottish terriers German shorthaired Pointers chihuahuas German shepherds Golden retriever (136)
Esophageal	Esophageal dysmotility	Border terrier West highland white terrier Manchester terrier (51)
Esophageal	Vascular ring anomaly	German shepherd (137–139) Greyhound (140) Irish setter (138) Labrador retriever (139)
Esophageal	Sliding (type I) hiatal hernia	Brachycephalic breeds (42) English bulldog French bulldog Boston Terrier Boxer Pug Chow Chinese Shar-Pei (141)

* *Indicates that these breeds get congenital megaesophagus secondary to congenital myasthenia gravis*.

Abnormal clinical signs of nasal reflux of milk or food, gagging, and retching immediately upon swallowing manifest shortly after birth, and are exacerbated when swallowing thin liquids compared to thicker liquids or solids. In contrast, cricopharyngeus muscle dysfunction in humans is a rare cause of pediatric swallowing impairment (96). Interestingly, cricopharyngeus muscle dysfunction is well-documented in elderly human patients and symptoms do not appear to be exacerbated following the consumption of liquids. The development of a cricopharyngeus bar, a radiologic descriptor of a posterior impression at the pharyngoesophageal segment, in elderly human patients as a compensatory mechanism to help prevent aspiration of refluxed material is an intriguing consideration; (97, 98) however, a cricopharyngeus bar is commonly observed during swallow fluoroscopy in young puppies diagnosed with cricopharyngeus muscle achalasia (92). Further research is warranted to elucidate the role of this radiologic descriptor in humans and dogs (Figure 4H).

**Figure 5:**
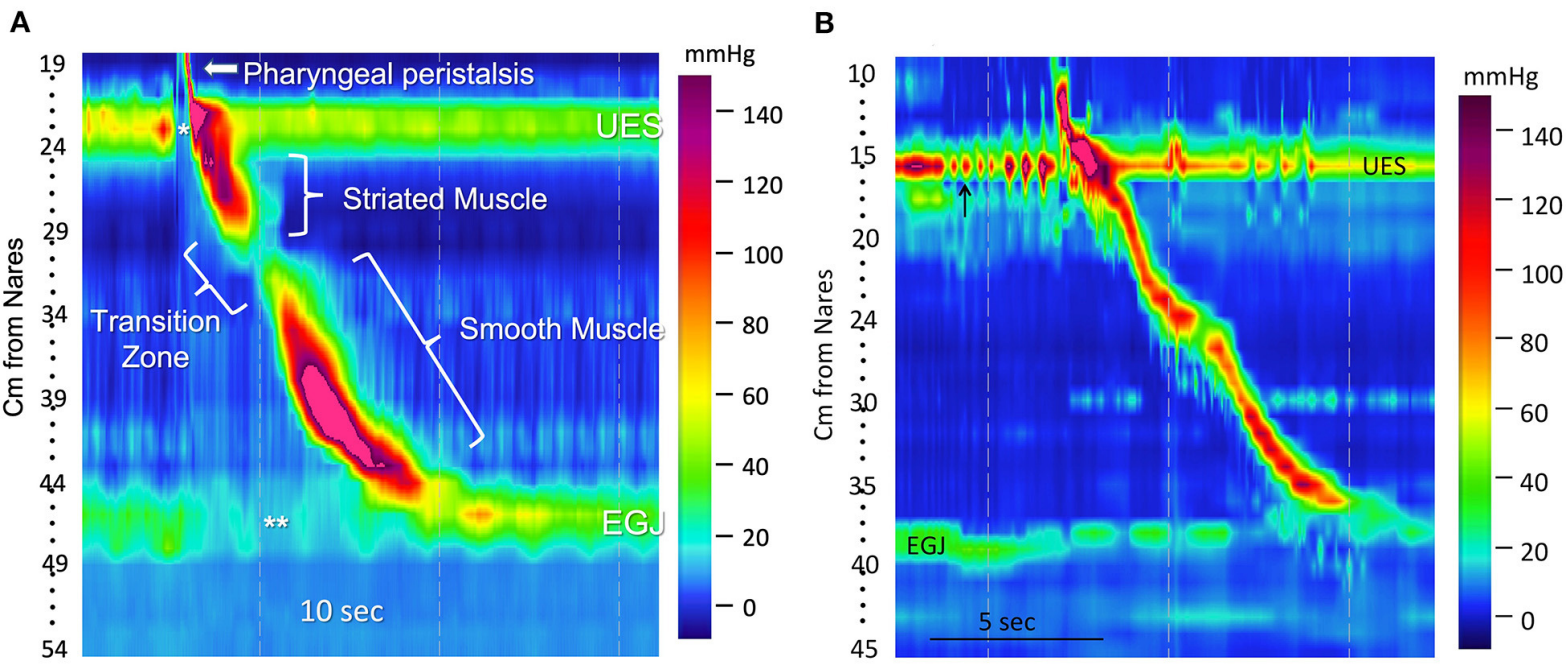
A transition zone is present in humans, but lacking in dogs. **(A)** A high-resolution color topographical pressure plot of esophageal motor function produced by a 5-milliliter water swallow in a human. It was obtained with a high-resolution manometry (HRM) catheter placed to simultaneously record pressures from the pharynx to the stomach. Pressure is represented by color coding (interpreted on the basis of the color bar on the right), sensor location (distance from the nares in cm) is on the y-axis, and time is on the x-axis. Resting UES (upper esophageal sphincter) and EGJ (esophagogastric junction) pressures are seen as horizontal bands of color that are several centimeters in width. Their hues indicate pressures that are greater than those in the adjacent portion of the pharynx, esophagus, or stomach. Opening of the UES (*) and LES (**) are depicted as changes of color to hues that represent a lower pressure. The narrow, diagonal bar of color above the UES in the pharynx (arrow) represents a pharyngeal contraction. A diagonal band of color running from the UES to 30 cm from the nares represents peristalsis of the striated muscle esophagus, and the diagonal band from 32 cm to the EGJ represents peristalsis in the smooth muscle esophagus. The area of diminished pressure separating these two bands denotes the transition zone over which the muscle is transitioning from striated to smooth. **(B)** A high-resolution manometry esophageal topography plot showing a pharyngeal contraction and esophageal peristaltic pressure wave generated by the swallow of a 5 g canned food bolus in a 7.2 kg terrier mixed breed dog. There is a continuous diagonal color band from UES to LES representing an uninterrupted peristaltic wave. This continuous peristaltic wave occurs because, except for the LES, the dog esophagus is striated muscle and lacks a transition zone. There are rhythmic contractions of the UES just prior to the swallow (arrow). The genesis of this contractile pattern is unclear, but might represent mastication. **(B)** was reprinted from American Journal of Veterinary Research, Volume 77, Ullal TV, Kass PH, Conklin JL, Belafsky PC, Marks SL, High-resolution manometric evaluation of the effects of cisapride on the esophagus during administration of solid and liquid boluses in awake healthy dogs, Copyright 2016 American Journal of Veterinary Research. Reprinted with permission from American Veterinary Medical Association.

Another difference in disease manifestation between the two species is in the prevalence of Barrett's esophagus (Figure 4J). Despite the high prevalence of GER in brachycephalics since birth and homology in LES anatomy, Barrett's esophagus and neoplastic transformation to adenocarcinoma rarely occurs spontaneously in dogs (99). Instead, the most common esophageal neoplasia in dogs is esophageal sarcomas (osteosarcoma, fibrosarcoma, chondrosarcoma, and undifferentiated sarcoma) secondary to the carcinogenic canine nematode, *Spirocerca lupi* (100, 101). Esophageal leiomyomas represent the most common benign tumor of the canine esophagus (Figure 4I). Disorders such as eosinophilic esophagitis and megaesophagus also affect the two species with differing prevalence. Eosinophilic esophagitis is far more prevalent in humans and is often diagnosed during childhood (102), however, there are only rare case reports in canines, despite the relatively high prevalence of food allergy, eosinophilic gastroenteritis, and atopy in dogs (48). This is juxtaposed by the high frequency of megaesophagus in dogs, which is diagnosed far more commonly in canines possibly due to differences in neuromuscular anatomy between the two species, and because of the far higher prevalence of myasthenia gravis in dogs. The canine esophageal body is composed entirely of striated muscle (35) whereas in humans, the proximal esophagus (up to one-third), including the UES, is striated and the distal third is smooth muscle with a transition zone between (37) (Figures 5A,B) (53). The peristalsis in the striated muscle of both species is controlled by vagal efferents arising from the nucleus ambiguus in the brainstem (66, 103). These vagal efferents synapse directly on striated muscle motor endplates, and release acetylcholine. Acetylcholine stimulates nicotinic cholinergic receptors, which causes striated muscle contraction. Peristalsis in the striated segment occurs because a pattern generator in the nucleus ambiguus sequentially activates vagal efferents such that the striated muscle motor units are activated in a craniocaudal sequence along the length of the esophagus (104, 105). Control of peristalsis in the smooth muscle esophagus is quite different. Vagal efferents that arise in the dorsal motor nucleus of the vagus innervate myenteric neurons present between the circular and longitudinal muscle layers. Myenteric neurons are the terminal motor innervation of esophageal circular smooth muscle. These neurons are either excitatory or inhibitory. The excitatory neurons release acetylcholine, which activates muscarinic cholinergic receptors to produce contraction. Inhibitory myenteric neurons contain nitric oxide synthase. Their activation causes the release of nitric oxide, which relaxes the lower esophageal sphincter and inhibits contraction of the esophageal circular smooth muscle (106). Nitric oxide plays a key role in the generation of peristalsis in the smooth muscle esophagus, since blocking its production abolishes peristalsis and LES relaxation, and achalasia in humans is the result of loss of nitric oxide synthase neurons (106–108). Despite these differences, manometrically recorded esophageal motor function in canines and humans is remarkably similar (53).

Nonetheless, neuromuscular differences impact disease phenotype. For example, acquired secondary megaesophagus is more commonly diagnosed in dogs, particularly secondary to myasthenia gravis that is diagnosed in 25% of dogs with megaesophagus (77). This may be because myasthenic autoantibodies preferentially target nicotinic receptors in striated muscle, which is found throughout the canine esophagus, but only the cervical esophagus in humans (109). In contrast, peristaltic defects of the transition zone uniquely occur in humans because dogs lack this anatomic region (110, 111). Similarly, myenteric plexopathies that cause smooth muscle disorders such as distal esophageal spasm or hypercontractile esophagus in humans have not been diagnosed in dogs to date (36, 112, 113).

**Figure 6:**
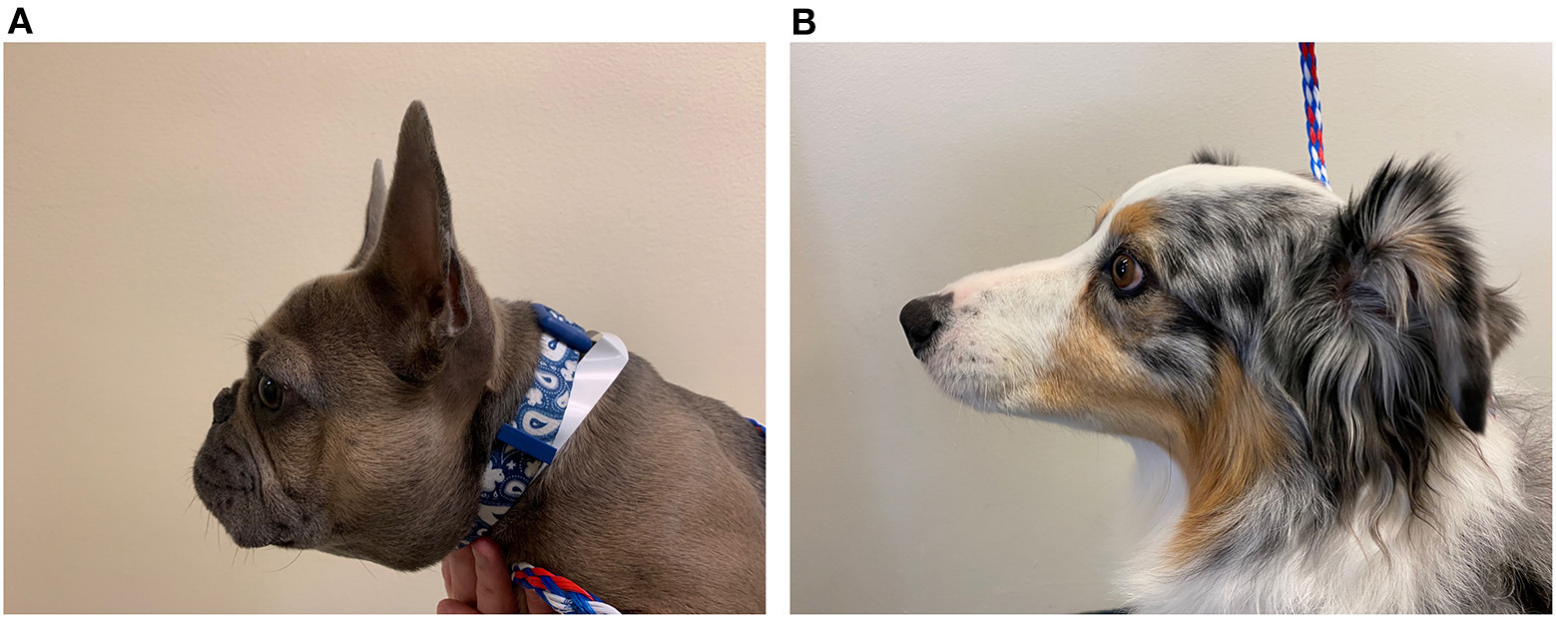
Brachycephalic vs. dolicocephalic conformation. **(A)** Picture of a brachycephalic 9-month-old French Bulldog showing the foreshortened muzzle, round face, and nasal folds in comparison to **(B)**. **(B)** Picture of a dolicocephalic breed dog, 7-year-old Australian Shepherd, with an elongated muzzle.

Other important anatomical differences between humans and canines include esophageal length and nasofacial conformation. Esophageal length in dogs ranges widely with the size and breed of the dog (114), ranging in length from ~20 to 70 cm compared to the consistent range of 18–26 cm in adult humans (115). Nasofacial structure also varies in dogs. Dolicocephalic or mesaticephalic breeds have longer noses and skulls and wider nares compared to brachycephalic breeds (116) (Figures 6A,B). This brachycephalic conformation makes placement of transnasal endoscopes, feeding tubes, and manometric catheters more challenging because their nares are small and their foreshortened muzzles are difficult to grasp during placement. However, the increased nasal length in dolicocephalic breeds can also complicate such procedures because the dead-space of the nasal passage can limit the endoscope or catheter from reaching the LES of the animal. Dogs also have more nasal turbinates compared to humans (117), which makes placing catheters and scopes transnasally more challenging.

Finally, the anatomy of a bipedal human predisposes to greater GER compared to quadrupedal dogs. Because humans stand upright, gravity assists with esophageal transit, but organs in the chest cavity apply a greater pressure on the diaphragmatic crura, which can compromise the gastroesophageal junction (GEJ). Additionally, the stomach in humans is positioned such that the antrum and pylorus are superior to the fundus whereas in dogs, the antrum is below the fundus to facilitate gastric emptying and minimize reflux (118). However, dogs appear predisposed to hiatal herniation compared to humans because they often lack an intra-abdominal esophageal segment. This exposes the thoracic esophagus and gastric cardia to intrathoracic pressures, which can result in hiatal herniation and reflux (119).

## Diagnostic Assessment of Swallowing Disorders in Canines and Humans

### Clinical Assessment

The approach to the human or canine patient with swallowing impairment begins with a careful review of the patient's signalment which is particularly important in the canine in light of hereditary and breed-related predispositions (Table 1) (7, 42, 51, 78, 89, 92–95, 120–141).

Congenital causes of swallowing impairment are commonly seen in young pure-bred dogs and several of these disorders are self-limiting in nature or can resolve spontaneously. Specific examples of the latter include juvenile esophageal dysmotility, a self-limiting disorder documented in terrier breeds <1-year of age that is thought to reflect delayed maturation of esophageal neuromuscular function (51). Congenital megaesophagus can resolve spontaneously although the probability of complete recovery is only 20–40% (142) despite higher rates of remission reported in miniature schnauzers (143). In contrast, swallowing impairment in adult or geriatric dogs is typically due to acquired neuropathies, myopathies, or junctionopathies. Clinical signs of regurgitation, hypersalivation, cough secondary to aspiration pneumonia, and emaciation are seen secondary to primary esophageal involvement, however, systemic manifestations of polyneuropathy (geriatric-onset laryngeal paralysis polyneuropathy) or polymyopathy can also be associated with weakness, ataxia, and fever (144).

The common etiologies of dysphagia differ depending on age group for humans as well. Congenital disease is more common in infancy. Traumatic brain injury and neck infections occur more often in pediatric patients (145). GER or immunologic causes (eosinophilic esophagitis or inflammatory myopathy) are more likely in children and adults. Esophageal achalasia more commonly affects the middle-aged. Neurodegenerative disease (Parkinson's disease, dementia, stroke) and neoplasia typically affect the elderly (145).

After considering patient signalment, obtaining a thorough medical history is essential in the assessment of a human or canine patient with swallowing impairment. However, obtaining a history from a human patient is more straightforward. Human patients can describe whether they experience regurgitation, reflux or heartburn, dyspepsia, globus, coughing, choking, drooling, aspiration, or concerning alarm symptoms (vomiting, gastrointestinal bleeding, weight loss) that will expedite diagnostic testing. They can convey the time course of their symptoms as chronic and intermittent (suggestive of a motility disorder) or rapidly progressive, which in combination with weight loss would raise concern for esophageal malignancy. They can indicate whether symptoms worsen with solid foods over liquids, which would suggest a mechanical obstruction caused by a stricture, ring, or web. They can also convey whether their symptoms occur within a second or two of swallowing, which would be suggestive of oropharyngeal swallowing impairment or a proximal esophageal web because the entire pharyngeal swallow occurs in this time domain. Difficulty swallowing that occurs in 5–10 s or more is consistent with esophageal dysfunction, because this is the time over which peristalsis travels to the esophagogastric junction (EGJ). Human patients can point to the area of their discomfort (subxiphoid, mid chest, or cervical) to help localize the problem. Finally, they can report medication and food/seasonal allergy history, which may reveal causative agents of pill-induced esophagitis or triggers for eosinophilic esophagitis, respectively (146). In contrast, canine patients cannot verbally communicate their history and clinical signs. Furthermore, pet owners can misreport regurgitation as vomiting or overlook subtle behaviors such as hard swallowing, lip smacking, and burping. Most pet owners do not closely observe their pets eating or drinking, and mild swallowing abnormalities can easily be missed. In addition, pets are often left unobserved at home during the day when owners are working, further increasing the challenges of obtaining a comprehensive and accurate history from the owner. Thus, veterinarians and pediatricians have similar challenges in assessing their dysphagic patients. To overcome these hurdles in communication, veterinarians must ensure they elicit comprehensive histories from the pet owner. An example list of history and clinical details a veterinarian should inquire about in the assessment of a canine with swallowing impairment is summarized below (147).

1. Age of onset (congenital vs. acquired)
2. Onset of swallowing problem (sudden vs. gradual)
3. Duration of signs (acute vs. subacute vs. chronic)
4. Frequency of signs (intermittent vs. persistent)
5. Progression of signs (static vs. progressive)
6. Temporal pattern (oropharyngeal swallowing impairment will occur within seconds of food or water consumption; esophageal swallowing impairment will occur seconds to hours following food or water consumption)
7. Associations with meals, activity (exacerbates hiatal herniation), or sleep (nocturnal GER)
8. Difficulty with solids, liquids, or both (canine patients with cricopharyngeus muscle achalasia typically experience exacerbation with liquids whereas patients with esophageal strictures experience exacerbation with solid foods)
9. Weight loss (weight loss from chronic regurgitation or reduced food intake) or weight gain (obesity can worsen GER)
10. Weakness, painful or stiff gait, exercise intolerance (suggestive of polymyopathy, polyneuropathy, or junctionopathy)
11. Dysphonia and dyspnea, history of laryngeal paralysis (suggestive of polyneuropathy, polymyopathy, or junctionopathy)
12. Recent administration of medications (pill-esophagitis or stricture formation secondary to clindamycin, doxycycline, tetracycline, ampicillin or non-steroidal anti-inflammatory drug administration)
13. Recent general anesthesia (causing GER and subsequent esophagitis or stricture formation)
14. Historical episodes of aspiration pneumonia (suggestive of aerodigestive disorders)
15. Change in diet (to identify dietary triggers of inflammatory bowel disease or eosinophilic esophagitis or to recognize increased dietary fat content that could precipitate delayed gastric emptying)
16. If brachycephalic dog breed, severity of brachycephalic obstructive airway syndrome (BOAS) and history of previous airway surgery (impacts management of hiatal herniation and GER).

Dysphagia questionnaires (148), such as the Eating Assessment Tool (EAT-10) (149), are used in humans to obtain history, score severity of disease, measure quality of life, and monitor treatment response. A Dog Swallowing Assessment Tool (Dog SAT) is a similar questionnaire currently being validated in dogs to assess the severity of swallowing impairment and help classify the anatomic localization of disease (43) (Supplementary Figure 1). However, the survey still depends greatly on the recognition of pet owners to accurately gauge the signs of swallowing impairment in their pets.

Given the challenges of obtaining comprehensive and accurate histories, a thorough physical examination of the canine patient with swallowing impairment is critical to augment the history and yield important clues. The physical examination should include an assessment of the oral cavity, throat and neck palpation, neurologic evaluation with cranial nerve tests and gag reflex, facial symmetry, muscle atrophy, body condition, and nutritional status. Finally, a critical aspect of the examination is observing the canine patient swallow, which can help characterize and localize the swallowing impairment.

In humans, physical exams may reveal weight loss and frailty, muscle atrophy, neurologic abnormalities, or specific dermatologic abnormalities indicative of connective tissue diseases (scleroderma). Bedside swallow tests (150) can be useful to assess which consistencies a human patient can tolerate (rheology assessment). They can also help screen at-risk neurologic or elderly patients for choking and aspiration. Specifically, poor hyoid elevation during a dry swallow and repeated throat clearing or a wet vocal quality after a wet swallow are suggestive of pharyngoesophageal dysfunction (98). Cognitive assessments and evaluations of social and emotional health can be especially important in the elderly with dementia (76). Psychological evaluations may also be indicated to investigate psychogenic or functional dysphagia if patients are suffering from globus or choking despite normal anatomy and swallow function (151, 152).

Even if there are no reported signs of swallowing impairment and swallow exam is normal, a history of recurrent aspiration pneumonia or chronic cough should alert both veterinarians and physicians to screen for aerodigestive disease (153, 154) and silent (subclinical) aspiration (155). Thus, although history and physical exam are important initial steps of the evaluation, further diagnostic testing with imaging or endoscopy are typically needed to identify aspiration and characterize the swallowing impairment accurately (146, 156).

### Contrast Radiography

Imaging in the canine patient typically begins with plain survey radiographs of the cervical region and thorax (3-views) to screen for anatomical and structural abnormalities, including megaesophagus (7, 157) (Figure 4E) (82), vascular ring anomalies (138), hiatal herniation (42, 43) (Figure 3A) (82), foreign bodies (Figure 4C) (82) or intra- or extra-esophageal masses. Radiography with contrast material can delineate strictures, esophageal mass lesions, perforations, and vascular ring anomalies. However, contrast enhanced swallowing fluoroscopy is the gold standard to diagnose swallowing disorders in dogs because it provides a real-time assessment of deglutition (2).

### Swallowing Fluoroscopy

#### Indications

The primary objectives of swallowing fluoroscopy are to localize the swallowing impairment and diagnose its etiology. Swallowing fluoroscopy can also detect tracheal aspiration or laryngeal penetration and guide the management of at-risk patients by modifying the diet consistency or specifically for human patients, teaching compensatory maneuvers.

In both dogs and people, structural abnormalities including strictures, vascular ring anomalies, foreign bodies, esophageal malignancy, and hiatal hernias or pharyngeal weakness, cricopharyngeus muscle achalasia, delayed opening of the upper esophageal sphincter, esophageal achalasia, esophageal dysmotility, and GERD can be diagnosed with swallowing fluoroscopy (3). Fluoroscopy can also confirm aerodigestive disorders in dogs and people with a respiratory history or signs (153, 154). However, there are several other esophageal disorders more commonly identified in humans. Examples include esophageal webs (thin, eccentric squamous epithelium membranes typically found in the proximal esophagus) or rings (thin extensions of tissue causing narrowing typically in the distal esophagus). Tissue in webs and rings is <3 mm in width compared to >3 mm in strictures (158). Other examples include spastic esophageal motility disorders such as distal esophageal spasm (112) or hypercontractile esophagus (113), viral and fungal infectious esophagitis (159), and numerous other causes of oropharyngeal (160) and esophageal swallowing impairment [diabetes mellitus (161), Alzheimer's disease (162), Parkinson's disease (163), Huntington's chorea (164), multiple sclerosis (165), and scleroderma] (16).

Swallowing fluoroscopy informs diet recommendations and safer feeding practices that can enhance swallow function and reduce aspiration risk in dogs and humans (3, 166). Duration of time spent upright in a Bailey chair to facilitate gravity-assisted feeding and treatment modifications can be advised for dogs with megaesophagus (41, 166). Follow-up fluoroscopy can also assess treatment responses or outcomes in dogs or humans that undergo hiatal hernia surgery or achalasia interventions (43, 167, 168).

#### Procedure

During the fluoroscopic study, patients consume liquid and food boluses of various consistencies mixed with radio-opaque contrast material. In dogs, liquid contrast followed by soft canned food and kibble mixed with barium or iohexol are given, which is analogous to barium liquid, barium pudding, and barium biscuits used in humans. As patients swallow, images are captured as digitized recordings by a fluoroscopy unit at a preferred rate of 30 frames per second. Each frame is then analyzed frame-by-frame to assess swallow kinematics (2, 3).

Although the basic protocol is the same in humans, adjunctive techniques are used in people to minimize aspiration, expose impairments, and formulate therapeutic recommendations (57, 169–172). Firstly, patients are evaluated in both the lateral and anterior-posterior views to maximize visualization and diagnostic yield. Secondly, the patient is asked to perform multiple swallow tasks with varying bolus volumes (5 mL amounts, clinician-directed sips, self-directed swallows), viscosities (thin liquid, thick liquid nectar, thicker liquid honey), food textures (pudding and shortbread cookie), and delivery mechanisms (cup and spoon) to improve diagnostic yield (169, 170). Also, different temperatures, carbonation, and flavors are sometimes altered to change the sensory experience (173–176). Each distinct swallow type provides unique and complementary information for a holistic assessment of the patient. The shortbread cookie is the ideal method to assess oral clearance whereas large volume thin liquid boluses tend to reveal abnormalities in oropharyngeal dynamics (170). Higher viscosity barium or the addition of thickening gum-based agents may reduce the risk of aspiration and penetration. However, several studies show thickening agents can lead to increased post-swallow pharyngeal residues (177–180). Sour liquids can improve pharyngeal delays in swallows (174). The protocol can be further tailored to the particular patient. Larger, thicker boluses can be avoided if pharyngeal clearance of thin 5 mL liquid boluses already appears poor (169). Infants can be bottle-fed with different temperature milk-formulations at varying paces (175, 181). To evaluate the esophagus in more detail, double contrast examination can be performed where the human patient ingests gas producing effervescent tablets followed by barium. This technique fills the esophagus with both air and contrast to delineate mucosal irregularities (182, 183). A barium tablet (e.g., E-Z-DISK contains 700 mg of barium sulfate and is 13 mm in diameter) may also be used to better elucidate a stricture (184, 185).

Compensatory treatment strategies can then be trialed during the study (186). Patients may be asked to wait 3 s before swallowing (187, 188) or to hold their breath while swallowing (supraglottic swallow) to improve coordination and protect the airway from aspiration, respectively (186, 189). Patients may also be instructed to engage in a more forceful swallow (190) or adjust their head or neck postures to facilitate a stronger pharyngeal contraction (191). Specific lingual exercises may be tested during the videofluoroscopy to rehabilitate patients with stroke or traumatic brain injury (192, 193). Provocative maneuvers such as the water siphon test (patient asked to drink water while rolling into a right posterior oblique position) (194), cough stimulation, or Valsalva maneuvers can increase the detection of GER (195).

#### Challenges and Limitations

**Figure 7:**
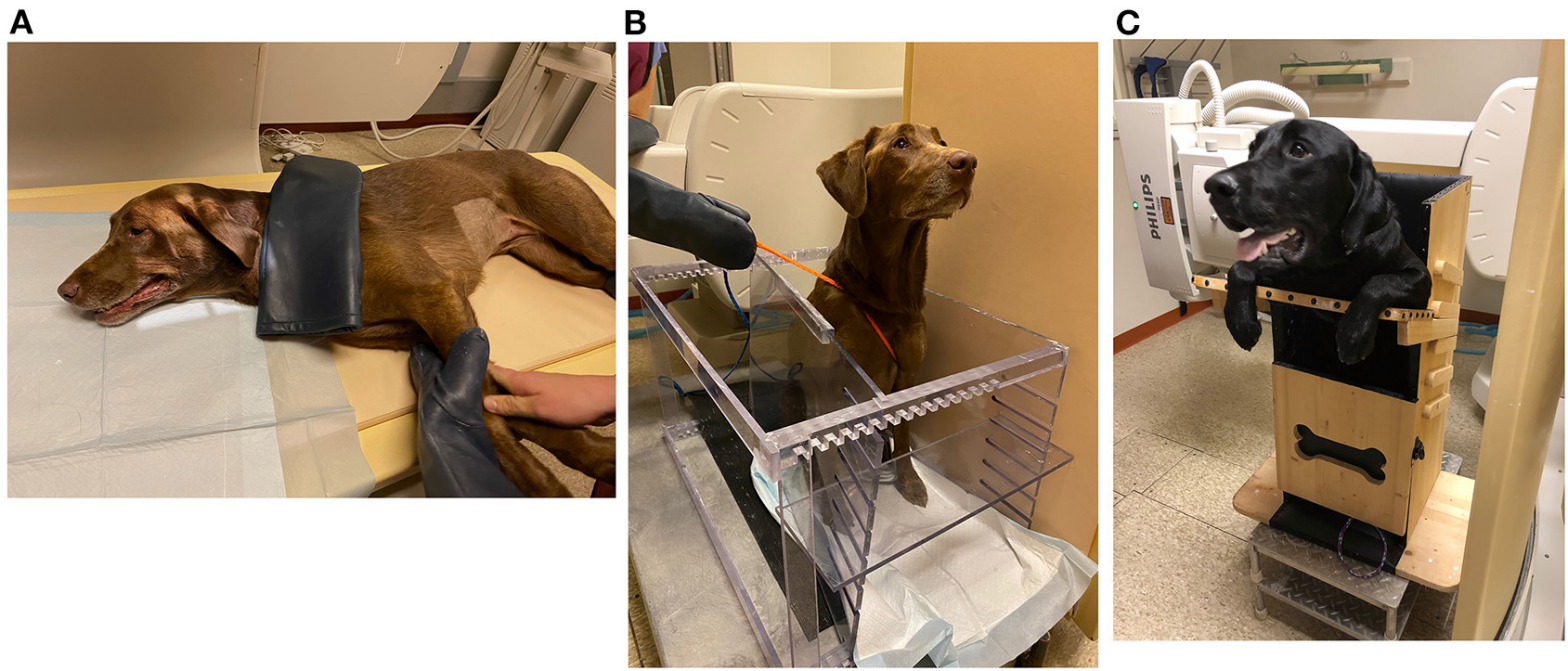
Positioning of dog in swallowing fluoroscopy study. **(A)** A 5-year-old Labrador retriever undergoing a videofluoroscopic swallow study examination in lateral recumbency with physical restraint by trained personnel. **(B)** The same patient in **(A)** undergoing a videofluoroscopic swallow study examination in a polycarbonate kennel restraint device. **(C)** An 11-month-old Labrador retriever undergoing videofluoroscopic swallow study examination in a Bailey Chair due to history of regurgitation. The Bailey Chair acts as a restraining device and maintains the dog in an upright position, enabling gravity to assist with passage of boluses down the esophagus.

Although swallowing fluoroscopy is the gold standard diagnostic procedure to evaluate swallowing impairment in dogs, there are many challenges to conducting the study in this species. Variables such as body position (196), bolus size, bolus type (197), use of physical restraint (198), sedation (199), and equipment can impact the study (172). Positioning of the dog in lateral recumbency is associated with delayed cervical esophageal transit and fewer primary esophageal contractions compared to a standing or seated position (196) (Figure 7A). Seated positions increase the hydrostatic pressure against the lower esophageal sphincter (41) and upright feeding protocols accelerate bolus transit time due to gravity (200) (Figures 7B,C). Solid boluses increase primary peristalsis, but delay pharyngeal contraction and slow esophageal transit compared to liquid boluses (197). Larger boluses shorten the time to UES opening compared to medium sized boluses (197). However, it is virtually impossible to have a dog swallow a consistent bolus volume despite best efforts to facilitate this practice. Even if specified volumes or amounts are administered, dogs may intentionally fragment the bolus into several swallows, particularly if doing so minimizes their signs of swallowing impairment. Large and giant-breed dogs are more likely to swallow the entire solid bolus rapidly without chewing, whereas toy-breed dogs are more likely to chew and fragment the bolus before swallowing resulting in marked variation in bolus size.

Patient factors such as the dog's size, temperament, severity of disease, and willingness to eat or drink can also affect study quality and accuracy. Non-compliant or larger breed dogs are difficult to physically restrain, which introduces motion artifact and exposes personnel to radiation. Polycarbonate kennel devices and Bailey chairs can restrain the dog and limit radiation exposure to personnel. These restraint devices can also facilitate free-feeding protocols during which the dog voluntarily consumes liquid and solid boluses (41, 166, 198). However, sedation could still be required to calm anxious dogs to facilitate the study, which can potentially alter esophageal motility (201) and sphincter tone (199). Furthermore, stressed, anorexic, or severely ill dogs may refuse to voluntarily prehend the bolus, requiring force-feeding practices that increase the risk of aspiration pneumonia or pre-empt a complete evaluation. Additionally, the compensatory treatment strategies used in humans require complex verbal directions that cannot be relayed to dogs. Dynamic disorders that occur intermittently such as GER or sliding hiatal hernia might also be missed given the limited duration of the swallow fluoroscopy study.

Due to these enumerable factors, swallowing fluoroscopy procedures are challenging to perform in dogs and difficult to standardize. Study protocols vary between patients and veterinary institutions (Table 2) (3, 41, 43, 51, 81, 153, 166, 168, 196–198, 202, 203). A standardization initiative amongst veterinary institutions and practices would improve reproducibility, but even with a uniform methodology, variability exists amongst healthy dogs and interpreting radiologists (172, 198). Optimally, objective swallow metrics such as inter-swallow interval, time to UES opening, time to maximum pharyngeal contraction, pharyngeal constriction ratio (204), and esophageal transit time would be used (198). However, further research of these parameters is needed to establish normative data and prove their diagnostic validity in dogs. In humans, there are a multitude of established swallow metrics in healthy individuals with normative data (205, 206). The effects of age (207), gender (208), bolus volume (209, 210), viscosity (173, 177, 178, 180, 209–211), carbonation, and palatability (176, 212) on these parameters are much better understood in humans. The swallowing reflex and UES opening delays with age (207) and larger bolus volumes (209, 210), higher viscosity (178), and carbonation decrease the risk of penetration and aspiration in some populations (176, 212). There are also many standardized study protocols and training systems such as the Modified Barium Swallow Impairment Profile (MBSImp) protocol (169) to minimize interoperator and interrater variability. However, even for humans, a comprehensive, evidence-based set of practice guidelines that is used globally is lacking (213).

**Table 2:** Swallow fluoroscopic study manuscripts in dogs.

	**Study population (number of dogs)**	**Prospective or retrospective**	**Body position**	**Restraint**	**Contrast type**	**Canned or kibble brand**	**Bolus size**	**Bolus number of each type**	**Qualitative or quantitative metrics**	**Feeding protocol**	**Sedation**	**Frame acquisition**
Effect of bolus size on deglutition and esophageal transit in healthy dogs Cheney et al. (197)	Healthy dogs (10)	Prospective	Right lateral recumbency	Physical restraint	60% w/v liquid barium sulfate	Purina Proplan EN Gastroenteric^†^	Liquid: 5, 10, 15 mL	≥3 swallows	Quantitative	Syringe-fed liquid	No sedation	30 frames per second
							Canned: 3, 8, 12 g			Offered or placed canned meatball in oral cavity		
Effects of body positioning on swallowing and esophageal transit in healthy dogs Bonadio et al. (196)	Healthy dogs (14)	Prospective	Right lateral recumbency and sternal	Physical restraint during lateral, polycarbonate kennel for sternal	60% w/v liquid barium sulfate	NR	Liquid: 5–10 mL	≥3 swallows	Quantitative	Syringe-fed liquid.	No sedation	30 frames per second
							Kibble: 5–10 kibble			Offered or placed kibble in oral cavity		
Quantitative videofluoroscopic evaluation of pharyngeal function in the dog Pollard et al. (81)*	Healthy (11) and dysphagic (3)	Prospective (healthy) Retrospective (dysphagic)	Right lateral recumbency	Physical restraint	60% w/v liquid barium sulfate	NR	Liquid: 10–15 mL	≥3 swallows	Quantitative	Syringe-fed liquid	No sedation	30 frames per second
							Kibble: NR			Offered or placed kibble in oral cavity		
Diagnostic outcome of contrast videofluoroscopic swallowing studies in 216 dysphagic dogs Pollard et al. (3)	Dysphagic (216)	Retrospective	Right lateral recumbency	Physical restraint	60% w/v liquid barium sulfate	NR	Liquid: 3–5 mL	≥3 swallows	Quantitative	Syringe-fed liquid	NR	30 frames per second
							Kibble: 5–6 kibble			Offered or placed kibble in oral cavity		
The prevalence of dynamic pharyngeal collapse is high in brachycephalic dogs undergoing videofluoroscopy Pollard et al. (202)	137: Dysphagic (89) or Cough (48) Brachycephalic (82) and non-brachycephalic (55)	Retrospective	Right lateral recumbency	Physical restraint	60% w/v liquid barium sulfate	NR	Liquid: 3–5 mL	≥3 swallows	Qualitative—pharyngeal collapse	Syringe-fed liquid	NR	30 frames per second
							Kibble: 5–6 kibble			Offered or placed kibble in oral cavity		
Standardization of a Videofluoroscopic swallow study protocol to investigate dysphagia in dogs Harris et al. (198)	Healthy dogs (24)	Prospective	Standing	Polycarbonate kennel	Liquid and puree: 25% iohexol	NR	Free fed	≥3 consecutive pairs of swallows	Quantitative	Free fed	NR	30 frames per second
					Kibble: barium sulfate 40% w/v							
Videofluoroscopic swallow study features of lower esophageal sphincter achalasia-like syndrome in dogs Grobman et al. (41)	Dogs with lower esophageal sphincter achalasia (19)	Retrospective	Standing and sitting	Polycarbonate kennel	Liquid and puree: 25% iohexol	NR	Free fed	≥3 consecutive pairs of swallows	Qualitative	Free fed	NR	30 frames per second
					Kibble: barium 40% w/v							
Aerodigestive disorders in dogs evaluated for cough using respiratory fluoroscopy and videofluoroscopic swallow studies Grobman et al. (153)	Signs of cough, but no esophageal or gastrointestinal signs (31)	Retrospective	Neutral standing or seated position	Polycarbonate kennel	Liquid and puree: 25% iohexol	NR	Free fed	≥3 consecutive pairs of swallows	Qualitative	Free fed	NR	30 frames per second
					Kibble: barium sulfate 40% w/v							
Esophageal dysmotility in young dogs Bexfield et al. (51)	Dysphagia (8), Healthy (22)	Prospective	Standing	NR	Canned food with barium sulfate (Polibar Rapid) 100.6% w/v	Fed patient's regular food	NR	Several	Qualitative	NR	4 cases sedated with IM acepromazine and buprenorphine 30 min before	NR
Retrospective analysis of esophageal imaging features in brachycephalic vs. non-brachycephalic dogs based on videofluoroscopic swallowing studies (50) Eivers et al.	Dysphagic brachycephalics and non-brachycephalics (36)	Retrospective	Standing	Physical restraint	Liquid barium undiluted	NR	Liquid not standardized	Not standardized	Qualitative	Syringe-fed liquid	Awake	NR
							Free fed			Free fed canned and kibble		
Prospective evaluation of surgical management of sliding hiatal hernia and gastroesophageal reflux in dogs Mayhew et al. (43)	Brachycephalics with dysphagia (17)	Prospective	Right lateral recumbency	Physical restraint	60% w/v liquid barium sulfate	NR	Liquid: 3–5 mL	≥3 swallows	Semi-quantitative	Syringe-fed kibble	Awake	30 frames per second
							Kibble not standardized			Offered or placed kibble in oral cavity		
Clinical and videofluoroscopic outcomes of laparoscopic treatment for sliding hiatal hernia and associated gastroesophageal reflux in brachycephalic dogs (168) Mayhew et al.	Brachycephalics with dysphagia (18)	Prospective	Right lateral recumbency	Physical restraint	60% w/v liquid barium sulfate	NR	Liquid: 3–5 mL	≥3 swallows	Semi-quantitative	Syringe-fed kibble	Awake	30 frames per second
							Kibble not standardized			Offered or placed kibble in oral cavity		
Technique for evaluation of gravity-assisted esophageal transit characteristics in dogs with megaesophagus (166) Haines et al.	Megaesophagus dogs (12)	Prospective	Upright in bailey chair	Bailey chair	60% w/v liquid barium sulfate	Purina ProPlan EN Gastroenteric^†^	Based on weight Liquid: 5, 10, or 15 mL	NR	Quantitative	Syringe-fed liquid, Unclear administration of canned and slurry	NR	NR
							Canned: 5, 10, 15, or 20 g					
							25% of slurry meal fed at home					

† *Purina® ProPlan® Veterinary Diets EN Gastroenteric™; Nestle Purina PetCare Company, St. Louis, Missouri*.

* *Same protocols in Inheritance of cricopharyngeal dysfunction in Golden Retrievers, Davidson et al. and Preliminary evaluation of pharyngeal constriction ratio (PCR) for fluoroscopic determination of pharyngeal constriction in dysphagic dogs, Pollard et al*.

*NR, not reported*.

#### Future Directions in Veterinary Medicine

Swallowing fluoroscopy methodology should be more standardized in veterinary practice. This will help establish quantitative normative data in dogs. Separate reference ranges for swallow metrics should also be established for specific age groups from juvenile to mature adult to geriatric dogs. This would optimize evaluation of oropharyngeal and esophageal function in animals of different ages. Although standardization is essential, study methodology should also be tailored to specific patients and clinical scenarios. In patients with cricopharyngeus muscle dysfunction, smaller volumes or thicker consistencies can minimize aspiration and improve swallow safety although larger volume, thinner consistencies can reveal abnormalities and improve diagnostic yield (178, 180, 210, 211). For dogs with esophageal achalasia, timed barium esophagrams can be performed in which barium retention is assessed at specified time intervals following ingestion (214, 215). For patients with suspected hiatal herniation or GERD, provocative maneuvers akin to those used in humans could be performed to improve detection (83). The stomach can be maximally distended with food or air to stimulate TLESR and subsequent GER. Patients can be rotated into different body positions to elicit abnormalities. Lateral recumbency will apply more pressure to the fundus to encourage hiatal herniation (114) whereas a seated position will increase hydrostatic pressure against a hypertonic LES in patients with esophageal achalasia (41). Thus, both standardization and individualization are important to improve the diagnostic utility of swallowing fluoroscopy in veterinary medicine.

The clinical applications of swallowing fluoroscopy extend beyond diagnosis. It can be utilized to assess outcomes of medical (sildenafil, botulinum toxin, pneumatic dilation) (167, 216) or surgical management (myotomy with fundoplication) (167) of esophageal achalasia, prokinetic and proton pump inhibition or surgical treatment of GER or hiatal herniation (43, 168), and laser or surgical myectomy for cricopharyngeus muscle achalasia (39).

### Fiberoptic Endoscopic Evaluation of Swallowing (FEES)

#### Indications

Fiberoptic endoscopic evaluation of swallowing (FEES) is a procedure commonly used in humans to evaluate oropharyngeal swallow function. FEES eliminates the use of specialized fluoroscopy equipment, contrast agents, and radiation required for swallowing fluoroscopy (217). Systematic reviews have shown an advantage of FEES over swallowing fluoroscopy to detect aspiration, penetration, and laryngopharyngeal residue in humans (218, 219). Guided observation of swallowing in the esophagus (GOOSE) can also be performed afterwards to identify a structural abnormality or evidence of delayed esophageal transit (220).

#### Procedure

The nasal passage is routinely anesthetized with 4–5 drops of topical anesthetic in dogs, and the tip of a fiberoptic endoscope (2.9 mm outer diameter in dogs) is lubricated before being passed transnasally in an awake patient. The endoscope is advanced until the scope is positioned between the soft palate and tip of the epiglottis, facilitating observation of the base of the tongue, vallecula, larynx, and both pyriform sinuses. The patient is then offered boluses of liquid and food stained with food coloring. During each swallow, laryngeal and pharyngeal anatomy is directly observed to evaluate the integrity of pharyngeal function and document evidence of penetration or aspiration (217, 221).

#### Challenges and Limitations

Disadvantages of the procedure are that the endoscope can interfere with deglutition and pharyngeal contraction can cause transient image white-out. Patients may also experience excessive gagging, coughing, or anxiety with scope placement. FEES was successfully performed without sedation in 6 healthy dogs; however, the procedure warrants further assessment in dogs with swallowing impairment. Additionally, passage of the 2.9 mm diameter scope may not be feasible in smaller dogs or brachycephalic breeds with narrow nares (221).

### High-Resolution Manometry

**Figure 8:**
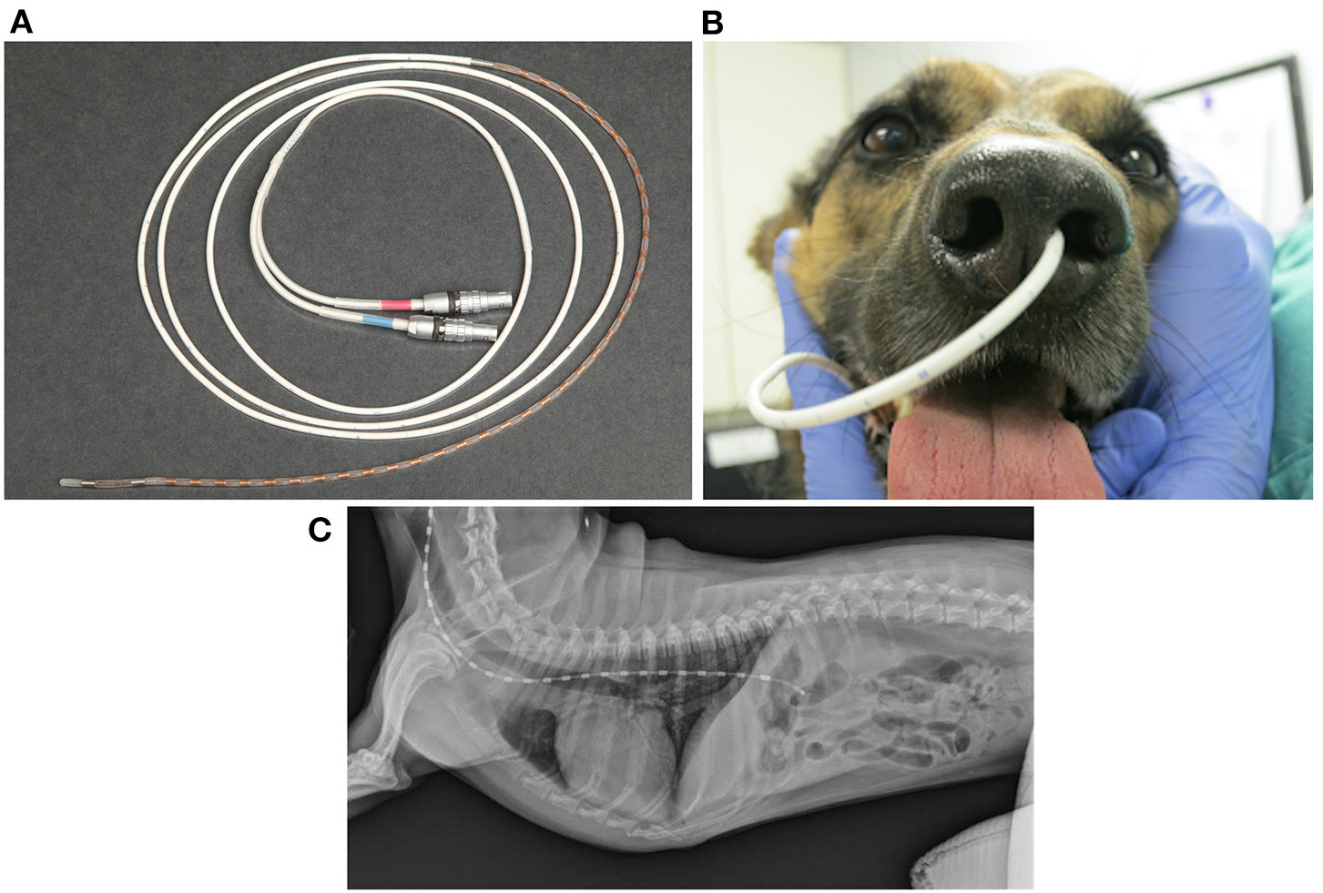
Implementation of high-resolution manometry in dogs. **(A)** A coiled 8Fr high resolution manometric (HRM) solid-state catheter with 36 circumferential pressure sensors spaced 1 cm apart, lining the end of the catheter. The red and blue labeled connectors plug into the manometry hardware module, which transmits information to the manometric data acquisition software that runs on a computer. **(B)** A picture of the HRM catheter successfully placed transnasally into the left nasal passage of a 4-year-old, 18-kg, mixed breed dog. **(C)** Survey lateral thoracic radiographic view of a 7.2-kg terrier cross after placement of a high-resolution manometry probe. In this dog, the probe traverses both the UES and LES. The brighter rectangular regions spaced equally along the probe represent each of the 36 probe sensors. **(C)** was reprinted from American Journal of Veterinary Research, Volume 77, Ullal TV, Kass PH, Conklin JL, Belafsky PC, Marks SL, High-resolution manometric evaluation of the effects of cisapride on the esophagus during administration of solid and liquid boluses in awake healthy dogs, Copyright 2016 American Journal of Veterinary Research. Reprinted with permission from American Veterinary Medical Association.

FEES or fluoroscopy is optimal to assess oropharyngeal dysphagia, but high-resolution manometry (HRM) is preferred to evaluate esophageal motility in humans (222). Esophageal manometry measures esophageal pressure profiles using an intraesophageal catheter lined with pressure sensors (223). Data captured at rest and during swallows are digitally converted to contoured line tracings or color topographical plots of pressure that depict esophageal motor function (Figures 5A,B) (53, 224). Compared to the 3–5 pressure sensors in conventional manometry catheters, high-resolution catheters have 36 pressure sensors spaced at 1-cm interval along the catheter, giving a 35-cm sensing segment. Each pressure sensor measures pressure at 12 positions around it circumference, which substantially improves the quantity and quality of data captured (225) (Figures 8A–C).

#### Indications

**Figure 9:**
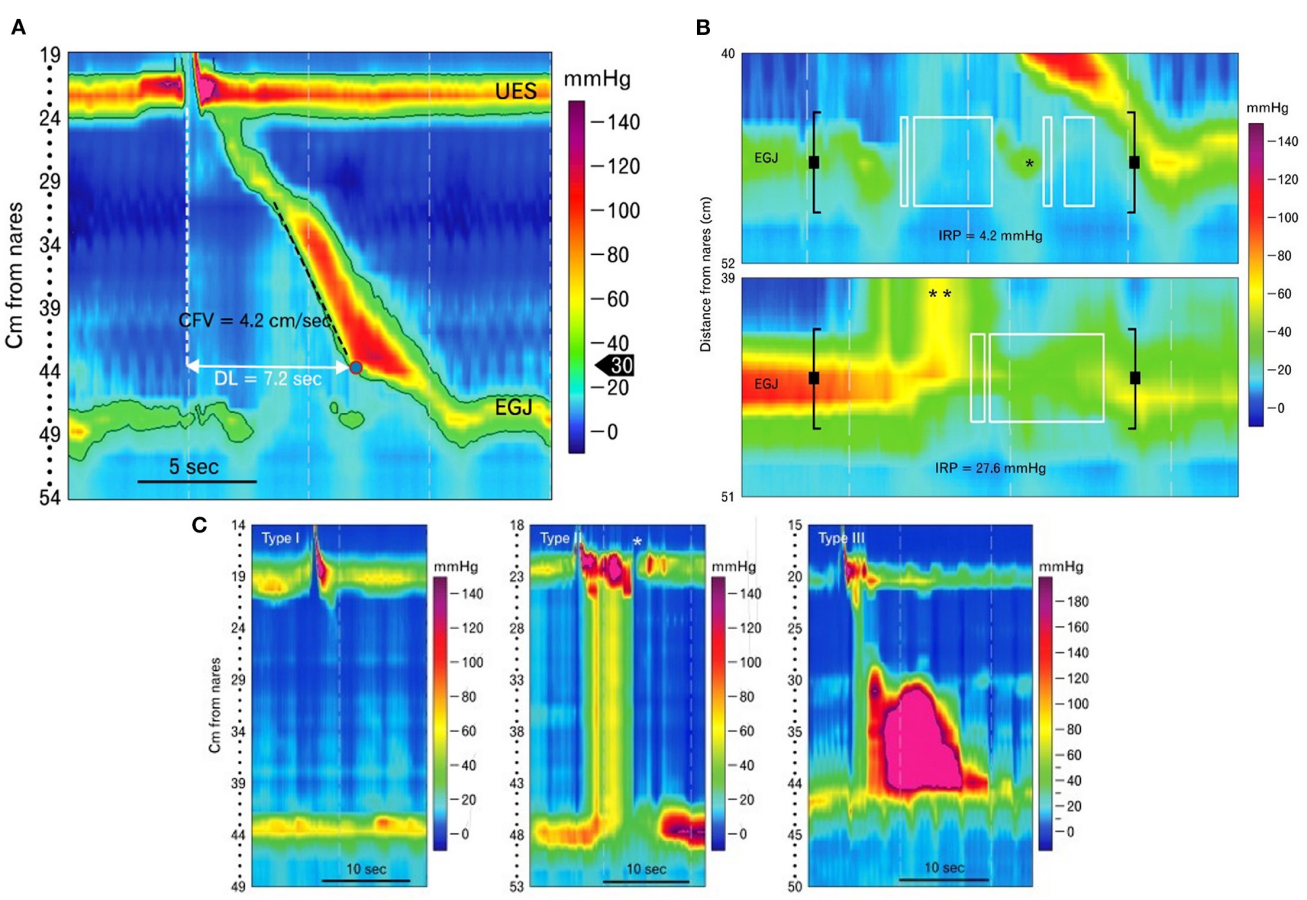
Esophageal pressure topography plots generated using high-resolution manometry in human patients. **(A)** High-resolution manometry catheters span and simultaneously measure pressures from pharynx to stomach and measure pressure throughout the esophagus. The results are graphically depicted in color contoured esophageal pressure topography plots as seen here. Metrics such as contraction front velocity (CFV) and distal latency (DL) can then be measured and calculated to evaluate esophageal peristalsis. CFV is a measure of peristaltic velocity in the smooth muscle portion of the esophagus. Distal latency is the time from upper esophageal sphincter (UES) opening to the contractile deceleration point (CDP), when peristalsis terminates at the esophagogastric junction (EGJ). **(B)** The top image shows the pressure topography of the esophagogastric junction (EGJ) following a liquid swallow in a normal human patient. Integrated relaxation pressure (IRP) is measured during a time window (bounded by black brackets) that occurs after upper esophageal sphincter (UES) opening. An eSleeve tool determines the highest pressure at each point in time within this window. An algorithm is then used to average the lowest of those pressures over four continuous or discontinuous seconds (marked by the white boxes). This average is the IRP. The example below is the EGJ pressure topography of a patient with achalasia. Due to failure of lower esophageal sphincter (LES) relaxation, excessive pressurization of the swallowed bolus occurs above the LES (**) and IRP is elevated. **(C)** Esophageal pressure topography plots showing the three different types of achalasia from left to right: Type I, II, and III. Achalasia is defined by a failure of normal peristalsis and lower esophageal sphincter opening and can be further classified into 3 types. Type I is characterized by absence of peristaltic activity in the esophagus without esophageal pressurization, type II by panesophageal pressurization and type III by premature esophageal contractions. The asterisk in the middle panel points to a brief opening of the UES that is not associated with a pharyngeal contraction or swallow and is therefore an example of the UES opening to vent. The images in this figure were published in Journal of Neurogastroenterology Motility; Volume 19, Conklin JL, Evaluation of esophageal motor function with high-resolution manometry, 281–294, Copyright 2013 by The Korean Society of Neurogastroenterology and Motility, Reprinted with permission from Editorial Office of Journal of Neurogastroenterology and Motility.

HRM is utilized to diagnose esophageal motility disorders in humans after obstructing lesions have been ruled out. Motility disorders can be classified into disorders of EGJ outflow including esophageal achalasia (type I, II, or III) and EGJ outflow obstruction or disorders of peristalsis such as absent contractility, distal esophageal spasm, hypercontractile esophagus, and ineffective esophageal motility (91, 226). The key metrics analyzed with HRM are integrated relaxation pressure (IRP), distal contractile integral (DCI), and distal latency (Figure 9A) (222) to assess LES relaxation, strength of esophageal peristalsis, and latency of deglutitive inhibition, respectively, which help characterize the type of major or minor motility disorder. For example, an elevated IRP denotes an esophageal outflow obstruction or esophageal achalasia (Figure 9B) (222). An increased DCI is supportive of a hypercontractile disorder such as jackhammer esophagus (hypercontractile esophagus). A short distal latency indicates premature contractions as seen in distal esophageal spasm (91). Interpreting IRP, DCI, and distal latency in combination aids in subtyping esophageal achalasia as type I, II, or III (Figure 9C) (222). Type I (classic) achalasia is marked by the absence of peristalsis in 100% of swallows, an elevated median integrated relaxation pressure (IRP > 15 mm Hg) and distal contractile integral (DCI) <100 mm Hg/s/cm; type II by panesophageal pressurization in ≥20% of swallows (most common subtype) and elevated median IRP (>15 mm Hg), and type III as elevated median IRP (>15 mm Hg) and premature, contractions in ≥20% of swallows with DCI > 450 mm Hg/s/cm (227).

An accurate diagnosis and classification optimizes treatment and informs prognosis. Esophagogastric junction (EGJ) outflow obstruction may resolve spontaneously or with discontinuation of opioid medications (58). Spastic disorders including distal esophageal spasm and type III (spastic) achalasia benefit from peroral endoscopic myectomy (POEM) surgery while type I or II achalasia have better outcomes with interventions focused on the LES (pneumatic dilation or Heller's myotomy of the LES) (228–231). HRM can also diagnose hiatal hernias (232) or evaluate esophageal motility before (233) and after anti-reflux fundoplication surgery (234, 235).

In contrast to human medicine, HRM is still a relatively novel procedure in veterinary medicine and has been utilized predominantly in healthy dogs. However, the procedure has been successfully conducted in awake dogs (53, 236, 237) and results showed vigorous peristaltic contractions with solid compared to liquid boluses and a significant increase in LES pressure induced by cisapride compared to metoclopramide (53, 236). Thus, with further study, HRM could improve the characterization and treatment of esophageal motility disorders in dogs.

#### Procedure

HRM can be conducted in both humans and dogs with a similar protocol except for a series of provocative maneuvers. The procedure begins with a temperature and pressure calibration. The calibrated manometric catheter is then placed transnasally in the awake patient. Humans should be positioned supine (12–30 degree angle) and dogs restrained in sternal recumbency or seated. The nasal passage is topically anesthetized with lidocaine jelly (238) ± a combination nasal spray of 1% tetracaine and 0.05% oxymetazoline (239) and the catheter is lubricated to facilitate passage of the flexible catheter into the nasopharynx and down the esophagus (Figures 8B,C) (53). Two to 3 mL of water can be administered orally to trigger peristalsis and advance the catheter past the LES. The distal 3–4 sensors are positioned intragastrically and the UES and LES should be visible on the image display as two bands of higher pressure above and below (Figures 5A,B) (53). Once the catheter position is set and the patient has acclimated to the catheter, baseline recording can begin. After baseline data is obtained, 5 mL boluses of water at room temperature are given and a swallow is recorded. This process is repeated until 10 consistent, intact swallows are obtained (227). In larger sized dogs and tall human patients with an esophageal length >30-cm, the manometry catheter may not span the entire esophagus, and the catheter is placed distally to span the distal esophagus and LES for acquisition of topographic data before being pulled proximally to repeat the procedure at the proximal esophagus, UES, and pharynx (53).

After the standard 10 water swallows, “provocative maneuvers” (240) are attempted in human patients to further test esophageal function. A multiple rapid swallow test (241) is performed by asking the person to swallow 5 times in quick successions. This helps reveal impairments in deglutitive inhibition or weak peristaltic reserve to diagnose achalasia (242) or ineffective esophageal motility (243), respectively. The individual is then moved to an upright position where at least 5 liquid swallows and another rapid swallow test are performed. The upright posture better replicates normal eating and improves detection of motility disorders (200) and hiatal herniation (244). If findings are equivocal, solid foods may be administered (245, 246) or post-prandial monitoring (247) is performed to improve diagnostic yield.

#### Challenges and Limitations

HRM has great potential in veterinary medicine, but there are many challenges to its implementation in dogs. Even with appropriate physical restraint of the patient, placement and retention of the manometric catheter is variably successful in the awake dog due to operator experience, patient non-compliance, and nasopharyngeal and esophageal anatomy. Dogs can be challenging to restrain during passage of the catheter transnasally, and are extremely sensitive to catheter insertion, particularly brachycephalic breeds with their shorter muzzle, upper airway obstruction, and respiratory distress. The catheter can be forcefully sneezed out due to nasal irritation, causing transient epistaxis and potential damage to the fragile pressure sensors along the catheter. In patients with megaesophagus or a sigmoid esophagus from esophageal achalasia, the catheter can coil and loop back on itself in the distended esophagus or meet physical resistance at the LES. Even if the catheter is placed successfully, it can be challenging to maintain in place, particularly while the dog is swallowing liquid or food boluses. Sedation can calm the dog to facilitate placement and retention, but sedatives or tranquilizers such as butorphanol and acepromazine can affect manometric parameters (237), and chronic opiate administration is known to affect esophageal function in humans (248).

Additionally, the provocative maneuvers applied in humans can be imitated in dogs, but are more challenging to replicate. For example, dogs can be kept in an upright position in a Bailey chair if they are amenable, but movement of their heads is uncontrolled and dogs with osteoarthrosis may be uncomfortable sitting on their haunches. Furthermore, this position does not emulate the physiologic feeding position of dogs as quadrupeds. Pre-determined bolus weights or volumes can be syringe-fed at specific intervals to perform a multiple rapid swallow test, but dogs may partially swallow the bolus at irregular intervals or retain material from multiple boluses in their mouths before swallowing at unpredictable times.

Furthermore, as with swallowing fluoroscopy, HRM methodology and data interpretation has not been standardized in dogs, whereas in humans there is a standardized protocol and consensus (the Chicago Classification v4.0) to diagnose and categorize esophageal disorders (91). In addition to the challenges of performing and interpreting HRM in dogs, the cost of manometry probes, hardware modules, and software costs is excessive for most veterinary practices and pet owners, and the procedures are not covered by veterinary insurance. The procedure is thus restricted to a few veterinary academic institutions in which it is predominantly utilized as a research tool.

#### Future Directions in Veterinary Medicine

Further evaluation of HRM in dogs with swallowing impairment is needed to develop a system analogous to the Chicago Classification. However, the many challenges encountered with HRM could impede its widespread application in veterinary practice. Meanwhile, the role of HRM in human medicine is expanding. Emerging applications in humans include the implementation of three-dimensional (3-D) HRM, in which there is a 9-cm segment lined with 12 pressure sensing loci and 8 radially dispersed pressure sensors at each locus, to evaluate pressure distributions at the pharynx, UES, and EGJ (249) or combination HRM-impedance technology to evaluate post-prandial TLESRs (250) or belching disorders (247). HRM-impedance technology is also being trialed in humans to assess bolus transit and post-residue swallows in patients with oropharyngeal (251) and esophageal dysmotility (252, 253).

Once metrics and normal reference ranges are established, HRM should be performed in dogs with swallowing abnormalities to appreciate the spectrum of esophageal dysfunction. Performing HRM in clinical patients could confirm esophageal motility disorders similar to those found in humans including ineffective esophageal motility, hypercontractile esophagus, distal esophageal spasm, and esophageal achalasia. HRM could also help differentiate the causes of oropharyngeal dysfunction or assist in the diagnosis of hiatal herniation and GER in dogs. Improved detection of these disorders will invariably improve treatment and patient outcomes.

### Intraesophageal pH Testing

Esophageal pH-metry and combined pH-impedance monitoring are the optimal procedures to diagnose and monitor GER. Esophagography, swallowing fluoroscopy (254), endoscopy and esophageal biopsies can reveal esophagitis (183), Barrett's esophagus, or peptic strictures secondary to reflux (255), but these methods lack sensitivity (256, 257) to detect reflux compared to pH monitoring (148, 149). pH-metry collects data over ≥24 h to survey acid reflux and the addition of impedance technology helps detect non-acidic and weak acid reflux (258, 259). In addition, multi channel intraluminal impedance and pH monitoring (MII-pH) measures changes in electrical impedance to determine composition, direction, and movement of the refluxate (260) to track gastric content moving orad or esophageal content moving aborad.

#### Indications

pH-metry is used in humans to diagnose GERD, classify phenotype as erosive or non-erosive reflux disease (NERD), and direct treatment. Human patients symptomatic for GER are initially treated with a proton pump inhibitor (PPI), but up to 33% of patients do not respond to a 2-week PPI course (261, 262). For PPI-refractory patients, upper endoscopy and ambulatory pH monitoring are recommended to evaluate for confirmatory evidence of GER. pH monitoring can also be used in conjunction with methods that assess gastric motility to assess whether delayed gastric emptying is exacerbating GER (20, 263).

In contrast, ambulatory reflux monitoring is understudied and underutilized in veterinary medicine. pH monitoring in dogs has mainly been performed under anesthesia with catheter-based techniques (29, 164–185). Numerous factors such as age (84, 264), sex (265, 266), breed (267), body size (268), type of surgery (84, 269), length of pre-operative fasting (85, 264, 270), body position (84, 269), anesthetic agents (morphine, acepromazine, inhalant gases) (271–275), and use of maropitant (276, 277), acid suppressant (omeprazole and esomeprazole) and prokinetic medications (metoclopramide and cisapride) (54, 276, 278–281) have been evaluated in association with peri-anesthetic GER. However, results have been variable and often conflicting possibly due to differing anesthetic protocols, definitions of reflux, and methods of pH measurement (Table 3) (45, 54, 84, 85, 264–283). For example, in some studies, increasing age and prolonged pre-anesthetic fasting were identified as risk factors for GER (84, 270), but were found to be protective in others (264). Changes in body position were associated with acid reflux in one study (269), but had minimal effect on GER in other studies (84, 275). Medications such as metoclopramide and omeprazole (280, 281) were initially found to reduce reflux under anesthesia, but subsequent publications refuted this claim (276). pH/impedance technology has now shown that PPIs only raise esophageal pH enough to mitigate acid reflux (54, 279), but cisapride significantly decreases both acid and non-acid reflux (54).

**Table 3:** Manuscripts assessing gastroesophageal reflux in dogs using pH monitoring.

**Reference**	**Authors**	**Year**	**Journal**	**pH technique**	**Definition of reflux**	**Conclusions**
Effects of atropine and glycopyrrolate on esophageal, gastric, and tracheal pH in anesthetized dogs (273)	Roush JK, Keene BW, Eicker SW, et al.	1990	Vet Surg	pH catheter	pH <4.0 or > 7.5 at any time	- Atropine and glycopyrrolate had no effect on esophageal, gastric, or tracheal pH
Gastro-esophageal reflux during anesthesia in the dog: the effect of preoperative fasting and premedication (85)	Galatos AD, Raptopoulos D	1995	Vet Rec	pH catheter	pH <4.0 or > 7.5 at any time	- Most reflux events were acidic - Prolonged fasting associated with increased reflux and gastric acidity
Gastro-esophageal reflux during anesthesia in the dog: the effect of age, positioning and type of surgical procedure (84)	Galatos AD, Raptopoulos D	1995	Vet Rec	pH catheter	pH <4.0 or > 7.5 at any time	- Increased age associated with increased reflux and increased acidity - Intraabdominal surgery associated with increased reflux episodes - No association with body position and tilt
Effects of preanesthetic administration of morphine on gastroesophageal reflux and regurgitation during anesthesia in dogs (271)	Wilson DV, Evans AT, Miller R	2005	Am J Vet Res	pH catheter	pH <4.0 or > 7.5 at any time	- Administration of morphine prior to anesthesia increased frequency of reflux in healthy dogs
Influence of halothane, isoflurane, and sevoflurane on gastroesophageal reflux during anesthesia in dogs (272)	Wilson DV, Boruta DT, Evans AT	2006	Am J Vet Res	pH catheter	pH to <4 or to > 7.5 for a period of ≥ 30 s	- Risk of developing reflux did not differ between anesthetic inhalants
Influence of metoclopramide on gastroesophageal reflux in anesthetized dogs (281)	Wilson DV, Evans AT, Mauer WA	2006	Am J Vet Res	pH catheter	pH to <4 or to > 7.5 for a period of ≥ 30 s	- High dose of metoclopramide (bolus 1.0 mg/kg IV, followed by CRI of 1.0 mg/kg/h) associated with a 54% reduction in relative risk of developing GER - Low dose (bolus 0.4 mg/kg IV, then CRI of 0.3 mg/kg/h) did not significantly affect GER
Pre-anesthetic meperidine: associated vomiting and gastroesophageal reflux during the subsequent anesthetic in dogs (274)	Wilson DV, Tom Evans A, Mauer WA	2007	Vet Anaesth Analg	pH catheter	pH to <4 or to > 7.5 for a period of ≥ 30 s	- Meperidine decreased risk of GER by 55% compared to morphine alone, but was not statistically significant and provided inadequate sedation
Effect of endogenous progesterone and oestradiol-17 beta on the incidence of gastro-esophageal reflux and on the barrier pressure during general anesthesia in the female dog (266)	Anagnostou TL, Savvas I, Kazakos GM, et al.	2009	Vet Anaesth Analg	pH catheter	pH <4.0 or > 7.5 at any time	- No significant differences in reflux between females with basal to high levels of estrogen and progesterone
The effect of omeprazole on esophageal pH in dogs during anesthesia (280)	Panti A, Bennett RC, Corletto F, et al.	2009	J. Small Anim Pract	pH catheter	Abrupt decrease to pH <4.0	- Group that received 1 mg/kg omeprazole at least 4 h prior to anesthesia had significantly less frequent reflux compared to control
Ambulatory esophageal pHmetry in healthy dogs with and without the influence of general anesthesia (275)	Favrato ES, de Souza MV, dos Santos Costa PR, et al.	2009	Vet Res Commun	pH catheter	pH <4.0 acid reflux, non-acid reflux identified by visualizing refluxate in esophagus with endoscope at end of surgical procedure	- Mean esophageal pH significantly lower in anesthetized vs. awake dogs - Minimal variation in esophageal pH in awake dogs, even with changes in body position
Evaluation of metoclopramide and ranitidine on the prevention of gastroesophageal reflux episodes in anesthetized dogs (278)	Favrato ES, Souza MV, Costa PR, et al.	2012	Res Vet Sci	pH catheter	pH <4.0 acid reflux, non-acid reflux identified by visualizing refluxate in esophagus with endoscope at end of surgical procedure	- Neither metoclopramide as bolus and CRI nor ranitidine bolus 6 hrs before anesthesia had any effect on incidence of GER under anesthesia
•The influence of esomeprazole and cisapride on gastroesophageal •Reflux during anesthesia in dogs (54)	Zacuto AC, Marks SL, Osborn J, et al.	2012	J Vet Intern Med	pH/impedance probe	50% decrement in ohms seen in 2 consecutive impedance channels for >2 s, classified as strongly acidic (pH <4.0), weakly acidic (4.0 < pH <7.0), or non-acidic (pH ≥ 7.0).	- Esomeprazole increased intraesophageal pH, but only combination esomeprazole + cisapride decreased frequency of GER compared to control
Maropitant prevented vomiting but not gastroesophageal reflux in anesthetized dogs premedicated with acepromazine- hydromorphone (277)	Johnson RA	2014	Vet Anaesth Analg	pH catheter	pH to <4 or to > 7.5 for a period of ≥ 30 s	- No significant differences in number of dogs that experienced reflux or reflux events between group that received maropitant pre-operatively and saline control
Wireless ambulatory esophageal ph monitoring in dogs with clinical signs interpreted as gastroesophageal reflux (45)	Kook PH, Kempf J, Ruetten M, and Reusch CE	2014	J Vet Intern Med	Bravo pH wireless capsule	pH <4.0 at any time	- No significant differences in esophageal pH or number of reflux events between healthy group and dogs clinical for reflux - Clinical sign-reflux association was poor amongst dogs clinical for reflux
The effect of the stage of the ovarian cycle (anoestrus or dioestrus) and of pregnancy on the incidence of gastro-esophageal reflux in dogs undergoing ovariohysterectomy (265)	Anagnostou TL, Savvas I, Kazakos GM, et al.	2015	Vet Anaesth Analg	pH catheter	pH <4.0 or > 7.5 at any time	- High incidence of reflux in female dogs in second half of pregnancy compared to dogs in anestrus or diestrus
Prospective controlled study of gastroesophageal reflux in dogs with naturally occurring laryngeal paralysis (283)	Tarvin KM, Twedt DC, Monnet E	2016	Vet Surg	pH/impedance probe	pH <4 (acidic reflux) or > 7.5 (alkalotic reflux) lasting for > 2 s reaching a minimum of 2 impedance sensors proximally along the probe	- Performed in awake dogs - Dogs with laryngeal paralysis had significantly more acidic reflux than normal controls
A “light meal” 2 h preoperatively Decreases the incidence of gastro-esophageal Reflux in dogs (270)	Savvas I, Raptopoulous D, Rallis T	2016	J Am Anim Hosp Assoc	pH catheter	pH <4.0 or > 7.5 at any time	- Significantly lower incidence of GER in dogs that received canned food 3 vs. 10 h before anesthesia
Gastro-esophageal reflux in large-sized, deep-chested vs. small-sized, barrel-chested dogs undergoing spinal surgery in sternal recumbency (268)	Anagnostou TL, Kazakos GM, Savvas I, et al.	2017	Vet Anaesth Analg	pH catheter	pH <4.0 or > 7.5 at any time	- Large-sized, deep chested dogs had significantly higher frequency of reflux compared to small-sized, barrel-chested dogs
Evaluation of gastroesophageal reflux in anesthetized dogs with brachycephalic syndrome (267)	Shaver SL, Barbur LA, Jimenez DA, et al.	2017	J Am Anim Hosp Assoc	pH catheter	Prolonged (> 20 sec) decreases (<4.0) or increases (> 7.5) in pH	- Controls had higher mean esophageal pH compared to brachycephalics, but no significant difference in % of GER
Prevalence of and risk factors for intraoperative gastroesophageal reflux and postanesthetic vomiting and diarrhea in dogs undergoing general anesthesia (269)	Torrente C, Vigueras I, Manzanilla EG, et al.	2017	J Vet Emerg Crit Care	pH catheter	pH <4.0 at any time	- Intraabdominal surgery, changes in body position, and length of anesthesia significantly associated with acid reflux
Effect of the duration of food withholding prior to anesthesia on gastroesophageal reflux and regurgitation in healthy dogs undergoing elective orthopedic surgery (264)	Viskjer S, Sjostrom L	2017	Am J Vet Res	pH catheter	pH <4.0 at any time	- Gastroesophageal reflux and regurgitation under anesthesia significantly associated with pre-anesthetic food withholding. Dogs that received light meal 3 h before anesthesia were 3x more likely to have reflux and 15x more likely to regurgitate (visible regurgitation from mouth during anesthesia) than dogs fasted for 18 h - Increased age associated with decreased risk of GER - dorsal recumbency associated with increased risk of GER
Evaluation of the effectiveness of preoperative administration of maropitant citrate and metoclopramide hydrochloride in preventing postoperative clinical gastroesophageal reflux in dogs (276)	Jones CT, Fransson BA	2019	J Am Vet Med Assoc	pH catheter	pH <4.0 or > 7.5 at a single time point, continuous data not obtained	- Dogs receiving maropitant subcutaneous 45 min before anesthesia and metoclopramide CRI did not have lower incidence of post-operative clinical reflux compared to control - Did not continuously monitor intra-operative reflux
Incidence of gastroesophageal reflux in dogs undergoing orthopedic surgery or endoscopic evaluation of the upper gastrointestinal tract (282)	Lambertini C, Pietra M, Galiazzo G, et al.	2020	Vet Sci	pH catheter	pH to <4 or to > 7.5 for a period of ≥ 30 s	- No difference in GER between acepromazine vs. methadone vs. butorphanol groups - No difference in GER between dogs undergoing endoscopy vs. orthopedic procedures
Effect of two different pre-anesthetic omeprazole protocols on gastroesophageal reflux incidence and pH in dogs (279)	Lotti F, Twedt D, Warrit K, et al.	2020	J Small Anim Pract	pH/impedance probe	- Decrease of impedance (at least 50% decrement in ohms) across 2 or more of the most distal impedance electrodes - GER pH calculated by averaging data points obtained every 5 s during each GER event - Classified as strongly acidic (pH <4.0), weakly acidic (pH ≥ 4.0 and <7.0) or non-acidic (pH ≥7.0)	- Two doses of omeprazole (first given evening before and second dose given 3 hrs before anesthesia) significantly decreased strongly acidic reflux compared to single dose of omeprazole and control - Single dose of omeprazole given evening before anesthesia had no effect

#### Procedure

**Figure 10:**
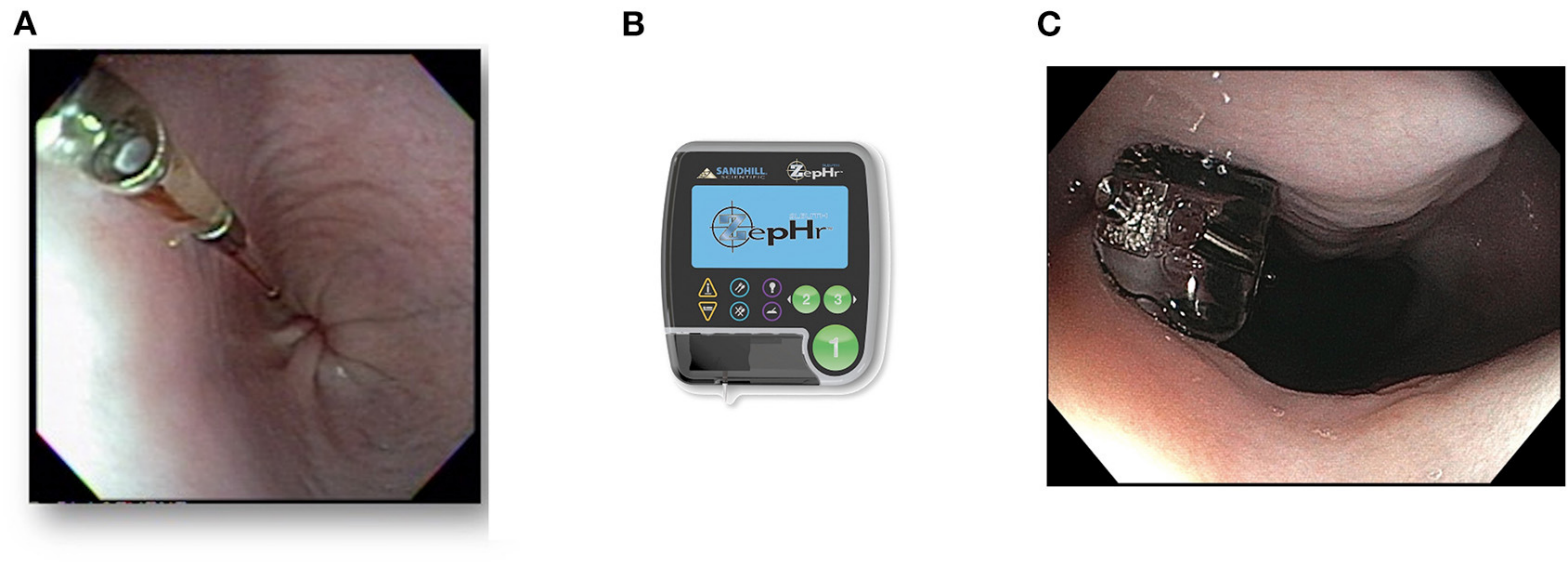
Multichannel intraluminal impedance/pH (MII-pH) catheter and ambulatory pH capsule. **(A)** Photographed is a 6.4-French (2.13 mm) esophageal multi-use impedance/pH probe in the esophagus of a dog patient. **(B)** The recording device (ZepHr) that the impedance/pH probe connects to. **(C)** A wireless Bravo^TM^ Calibration-Free Reflux pH Capsule tethered to the esophageal mucosa after placement in a 2-year-old French Bulldog with a diagnosis of hiatal herniation and gastroesophageal reflux.

The multichannel intraluminal impedance/pH catheter (MII-pH) is a 2.13 mm (6.4Fr) diameter catheter made of polyurethane. It has 6–8 impedance sensors, spaced 2-cm apart, and 1–2 pH sensors. After calibration in the appropriate pH buffer solutions, the catheter is placed transnasally or transorally (for assessment of GER in anesthetized canine patients) into the esophagus such that the proximal pH sensor is 5-cm above the LES or 6-cm proximal to the EGJ (Figures 10A,B). The catheter is affixed to the patient's face or around the dog's muzzle and kept in place for the duration of the study. Catheter-free ambulatory pH monitoring can be performed with a wireless Bravo pH capsule. Although impedance data cannot be obtained, pH data can be collected for up to 96 h, which minimizes variance and increases diagnostic sensitivity (284). The pH capsule is deployed with endoscopic guidance and tethered to the esophageal mucosa 6-cm proximal to the EGJ with a suction and locking pin mechanism (Figure 10C). The capsule measures pH every 6 s and transmits data to a receiver every 12 s using radio telemetry. Within 5–7 days, the capsule naturally detaches from the esophagus and passes through the intestinal tract. Data from the receiver can then be uploaded to a computer software program for analysis (285, 286). The primary outcome measure assessed is distal esophageal acid exposure time (AET). Acid exposure time >6% denotes pathologic GER, and <4% is considered physiologic in humans. Symptom association indices are also evaluated to determine the correlation of reflux events and patient reported symptoms of heartburn and regurgitation (255).

#### Challenges and Limitations

pH testing should be utilized more frequently in veterinary medicine to evaluate GER in non-anesthetized canine patients. Unfortunately, transnasal placement in an awake dog and retention of the probe for 24 h is challenging. However, ambulatory pH monitoring utilizing the wireless pH capsule (Bravo) is catheter-free and enables continuous pH data collection in the awake patient for up to 96 h. Results can show daily fluctuations in pH in association with the patient's symptoms, meal or water intake, body position, and activity (284, 287). As previously mentioned, the major limitation of wireless pH capsule monitoring is that it lacks impedance technology and therefore cannot diagnose non-acid reflux events or belching/rumination (repetitive regurgitation of undigested food due to a learned behavior) disorders. Additionally, minor technical complications such as breaks in data transmission, early dislodgement, or capsule retention can occur. However, non-trivial complications including esophageal wall trauma or capsule aspiration are rare (288). Another drawback to placing pH capsules in dogs is that the procedure requires general anesthesia and optimal positioning for placement is reliant on human studies. In humans, the capsule is consistently placed 6-cm proximal to the EGJ, but dogs have variable esophageal and sphincter lengths (114). Thus, a standard 6-cm distance may not be applicable to all dogs and could contribute to variation in pH readings. Another significant limitation to using pH monitoring in dogs is that normative data and metrics for analysis have not been established. In a study that used nuclear scintigraphy to evaluate reflux in healthy dogs, reflux events occurred on average twice every 5 min, however, pH monitoring was not performed to discern whether these were acid reflux events (289). A study that performed ambulatory pH monitoring in 7 healthy dogs documented a median of 10 acid reflux events (range 1–65) over a median study duration of 45 h (45).

In contrast to canines, there are conclusive criteria to diagnose GERD in humans. These have been summarized and published in the Lyon Consensus of 2018. They include both endoscopic criteria and metrics such as number of reflux episodes per 24 h, AET, SI (symptom index), and SAP (symptom association probability) (255). These metrics can even be assimilated into a DeMeester composite score (290) which directly correlates with endoscopic findings of erosive esophagitis. Endoscopic findings are even graded with established classification schemes such as the Los Angeles Classification system (291).

For example, if advanced grade erosive esophagitis (Los Angeles classification grade C and D) (Figure 4D), long-segment Barrett's mucosa, or peptic strictures are seen on endoscopy or distal esophageal AET > 6% on ambulatory pH or pH-impedance monitoring in a human patient, GERD is confirmed (255). However, <50% of human patients have endoscopic evidence of esophagitis (292, 293). These patients may have non-erosive reflux disease (NERD) or functional esophageal disorders.

If endoscopic findings are normal, but AET is >6%, NERD is diagnosed. If endoscopy and AET are both normal, further testing with pH-impedance is warranted to document non-acid reflux. Additionally, symptom-reflux association metrics SI (294) and SAP (295) should be analyzed. SI is the percentage of symptom events preceded by reflux episodes and SAP is the probability that symptoms and reflux events are associated. Patients with SI > 50% and SAP > 95% are predictive of better responses to medical and anti-reflux surgical therapy (296, 297). pH monitoring can also be repeated after anti-reflux surgery to assess surgical response. Symptom-reflux association is also used to diagnose functional esophageal disorders of altered nociception. Human patients with normal endoscopy and AET, but high symptom-reflux association may have reflux hypersensitivity. Alternatively, if symptom-reflux correlation is poor, functional heartburn is possible and cognitive behavioral therapy may be necessary (255).

The Lyon consensus also recognizes the heterogeneity of GERD in humans. For example, although an AET <4% is considered normal and > 6% defines pathologic reflux, patients may be diagnosed with borderline or inconclusive GERD based on low-grade esophagitis on endoscopy, equivocal AET between 4 and 6%, or normal AET but positive symptom-reflux associations.

In such cases, further testing with impedance and HRM may be warranted to interrogate for non-acid reflux, evaluate esophageal mucosal permeability, assess LES tone, and screen for poor esophageal contractility and delayed acid clearance (255). Novel impedance metrics including baseline impedance (298, 299) and post-reflux swallow-induced peristaltic wave (PSPW) index (300) can be used to assess esophageal mucosal integrity and peristalsis following a reflux episode, respectively. Although normative values for these impedance metrics are not yet available in humans, low baseline impedance is indicative of alterations in intercellular space and tight junctions secondary to reflux, and abnormal PSPW reflects diminished peristalsis and prolonged acid clearance (298, 301).

#### Future Directions in Veterinary Medicine

Additional pH/impedance studies should be performed in awake, healthy dogs to establish normative reference ranges for key metrics. This will provide a contextual basis to perform studies in clinical canine patients that help differentiate physiologic reflux, pathologic reflux, and functional/hypersensitivity conditions. A relevant patient population to study would be brachycephalic dogs given the high prevalence of GER and hiatal herniation (42, 50). Dogs with signs of aerodigestive disease would also be a pertinent patient group. The wireless Bravo pH capsule could help evaluate whether cough or nasal symptoms were correlated to reflux events. Once a classification system is developed, pH monitoring can be used in dogs, as it is in humans, to diagnose GERD phenotype, direct therapy, and assess response to medical or surgical treatments.

### Esophageal Histopathology

Further confirmation of GERD can be obtained by identifying esophageal histopathologic alterations secondary to GER. Examples include Barrett's esophagus (columnar metaplastic change) (302), dilation and edema of intercellular spaces, infiltration of mononuclear cells, proliferative basal cell hyperplasia, and papillary elongation of the squamous epithelium. Acute or healed erosions may also be seen (303, 304). Identification of such pathology can support the diagnosis of GERD (305) and serial biopsies can confirm treatment response.

In human patients, microscopic esophagitis significantly improves in response to administration of PPIs (306) and anti-reflux surgery (307). Similar findings have been observed in dogs (308, 309). Seventeen of 65 (26%) dogs clinical for reflux had evidence of hyperregeneratory esophagopathy (HRE), characterized by basal cell hyperplasia and papillary elongation, of which 12/17 (71%) responded positively to PPI treatment (46). Thus, esophageal histology not only aids in the diagnosis of GER, but justifies therapy. However, challenges to performing and interpreting esophageal biopsies in dogs should be recognized. Firstly, it is difficult to obtain endoscopic biopsies of the esophageal mucosa in dogs because the tissue is incredibly resilient. As a result, veterinary laboratories rarely receive adequate tissue samples for analysis. Even if a sufficient sample is obtained, normal esophageal histology does not exclude GERD (310, 311). Finally, there are no standardized criteria or established scoring systems to evaluate esophagitis in dogs.

In human patients with cricopharyngeus muscle dysfunction, biopsy of the cricopharyngeus muscle can be informative. Myositis of the cricopharyngeus muscle may occur secondary to polymyositis, dermatomyositis, or inclusion body myositis. Concurrent fibrosis indicates chronicity of disease (312, 313). Cricopharyngeus muscle histology in 5 dogs diagnosed with cricopharyngeus muscle dysfunction showed myofiber degeneration and atrophy suggestive of an underlying neuropathy, but larger sample sizes are needed (39). Severe ganglionic cell depletion of the LES in human patients with esophageal achalasia indicates progressive, end-stage disease, characteristic of type I achalasia (314); however, histopathology of the LES in dogs with esophageal achalasia-like syndrome has not been performed to date.

### Electrodiagnostics

#### Indications

Electrodiagnostic testing, including electromyography, nerve conduction velocity testing, and repetitive nerve stimulation, can confirm neuromuscular causes of swallowing impairment (315–317). Results can also guide selection of muscle and nerve biopsy sites (318). In humans, electromyography can also be used as a screening tool for dysphagia (319) and as biofeedback to guide swallowing rehabilitation (320). In dogs, electrodiagnostics have elucidated esophageal physiology (321) and explored pathology in patients with laryngeal paralysis (322) and megaesophagus (323–325). This has led to the hypotheses that vagal afferent dysfunction and secondary alterations in biomechanical properties explain the pathogenesis of idiopathic megaesophagus in dogs (323–326). Electromyography has also played an integral role in facilitating the diagnosis of inflammatory myopathies, including polymyositis, an immune-mediated disorder well-documented in Boxers and Newfoundlands (134).

#### Procedure

Electromyography is performed by inserting electrodes into skeletal muscles of interest such as pharyngeal, laryngeal, esophageal, thoracic, and pelvic limb musculature. Abnormal spontaneous electrical activity, characterized by scattered fibrillation potentials, positive sharp waves, and complex repetitive discharges, is consistent with a myopathy or neuropathy. Motor and sensory nerve conduction velocity testing and repetitive nerve stimulation measure electrical activity in muscles following nerve stimulation (316). Muscle biopsies can then be obtained to analyze affected myofiber types and identify features of inflammation, atrophy, or necrosis. Immunofluorescence staining on muscle biopsies can also characterize antibodies, major histocompatibility complexes, or T-lymphocytes to suggest polymyositis or identify protein deficiencies associated with muscular dystrophy (134). Nerve biopsies can be analyzed for inflammatory infiltrate, axonal degeneration, axonal dystrophies, demyelination, and nerve regeneration (318).

#### Challenges and Limitations

In humans, conventional electromyography electrodes can be placed intramuscularly in awake patients. Surface electromyography with adhesive electrodes (319) or skin patches can also be performed (320) in awake patients and can characterize swallowing disorders (327). In dogs, general anesthesia is required, which makes the procedure more costly and labor-intensive. Furthermore, procedural technique must be adhered to strictly to limit confounding variables. Electrodes must be grounded properly and inserted at various depths and locations in the muscles. Motion artifact must be minimized and the temperature of the muscle should be held constant because varying temperatures can affect readings (316). These requirements and specifications make these procedures complex and challenging to perform in veterinary practice.

### Endolumenal Functional Lumen Imaging Probe (EndoFLIP)

**Figure 11:**
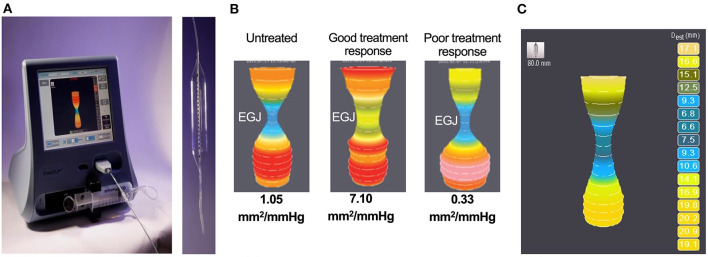
Endoluminal Functional Lumen Imaging Probe (EndoFLIP). **(A)** Image of the Endoluminal Functional Lumen Imaging Probe (EndoFLIP) machine and catheter with a soft balloon at the distal end. The EndoFLIP system uses impedance planimetry to map out the geometry of cross-sectional areas of the esophagus and esophagogastric junction (EGJ). **(B)** Intra-operative EndoFLIP images assessing the EGJ in human patients with achalasia pre and post Heller myotomy. The left image shows a patient pre-treatment with a narrowed EGJ. The middle image shows a patient with a good response to myotomy with an improved EGJ diameter. The right image shows a patient with a poor response to myotomy given the persistently narrowed EGJ. **(C)** Hourglass shape image generated by the EndoFLIP balloon catheter spanning the EGJ in a brachycephalic dog with a history of regurgitation. The numbers on the right indicate the diameter (in millimeters) read out at each 1 cm mark along the length of the 8 cm balloon.

Endolumenal functional lumen imaging probe (EndoFLIP) employs high-resolution impedance planimetry to analyze the cross-sectional area and distensibility of the esophagus and EGJ (328, 329). The EndoFLIP balloon catheter has 16 pairs of impedance electrodes and a single pressure sensor at the distal end (Figure 11A). The balloon portion of the catheter can be volumetrically distended to measure cross-sectional area and pressure at a given location. If that location is centered on the LES, an esophagogastric junction distensibility index (EGJ-DI) can be calculated by dividing the narrowest cross-sectional area by the intra-balloon pressure (330, 331). FLIP 1.0 measures EGJ-DI, but second generation FLIP 2.0 also evaluates esophageal motility with pressure topography. The distension of the balloon catheter triggers secondary peristalsis. The peristaltic contractions that occur can be classified into four different patterns: normal repetitive anterograde contractions, abnormal repetitive retrograde contractions, absent contractility, or other/diminished contractions (332, 333).

#### Indications

EndoFLIP has numerous indications in humans, but the primary use is to evaluate distensibility of the EGJ for pathology. EndoFLIP can complement or further support HRM and esophagography, particularly for patients in whom results are mismatched or equivocal (333, 334). An abnormally low EGJ-DI is expected in human patients with EGJ outflow obstruction or achalasia as compared to an elevated EGJ-DI in patients with GERD (331, 335). However, FLIP can also be used intra-operatively or post-operatively in achalasia and GERD patients to tailor surgical procedures. Real-time intra-operative data can guide surgeons in adjusting the extent of the myotomy or tightness of the fundoplication wrap (Figure 11B). Post-operative DI can then be compared to pre-op and intra-operative data to assess surgical outcome and predict clinical outcome (218–221). FLIP can also be used to measure stricture dimensions and guide balloon dilation in patients (336). This is particularly helpful for strictures in the pharyngoesophageal region because this area can be difficult to visualize endoscopically (337). In human patients with eosinophilic esophagitis, FLIP can monitor disease activity and track fibrotic remodeling that can occur with chronic inflammation (338). A less common indication of FLIP is to diagnose hiatal herniation by identifying a double-sphincter image caused by the separation of the LES from the crural diaphragm with the hiatal hernia in between (339). EndoFLIP could theoretically be used for the same indications in dogs; however, only EndoFLIP 1.0 has been evaluated in dogs to date in brachycephalic breeds following hiatal hernia surgery (Figure 11C) (43, 168).

#### Procedure

The EndoFLIP balloon catheter is placed transorally under sedation or light anesthesia. The catheter is positioned with a couple sensors in the stomach and the mechanical pump fills the balloon to a volume of 20 mL with an electrolyte solution of known conductance. At this point, the EGJ should be visualized as a narrowing of the hourglass shape on the image display. After 15–30 s, additional 10 mL aliquots of volume are instilled with wait periods of 30–60 s between until the balloon is distended to the recommended volume (70 mL). The volumetric distension should trigger secondary peristalsis for the contractile pattern to be observed with EndoFLIP 2.0. EGJ-DI can also be assessed once the volume is at least 60 mL and pressure is at least 15 mmHg. After all measurements are obtained, the balloon is deflated and the catheter is removed (329).

#### Challenges and Limitations

Although EndoFLIP is an attractive technological modality, there are a few barriers to entry in veterinary medicine. Firstly, the cost of EndoFLIP 2.0 equipment costs ~$70,000, excluding the cost of the single-use catheters ($350 each) (329). In the study of brachycephalics undergoing open hiatal hernia surgery, significant changes to EGJ geometry and DI were not found. Furthermore, the characteristic double-sphincter view indicative of a hiatal hernia was not observed in any of the dogs pre-operatively (43). Brachycephalic breeds most commonly have type I hiatal herniation, which is intermittent and dynamic (43, 168). Thus, measuring EGJ-DI at static, set volumes and time-points may not be adequate to detect sliding herniation. The mechanics of the EGJ are also incredibly complex, involving the hiatus, phrenoesophageal ligament, crural diaphragm, angle of His, and the LES (36). By obtaining measurements intraluminally, EndoFLIP may not be capturing all the relevant exatraluminal components and pathophysiology. It is also unclear whether distension of the EndoFLIP 2.0 balloon will stimulate the same contractile patterns in dogs because the musculature of the distal esophagus is different from that in humans (35). Another potential drawback to EndoFLIP is the patient must be anesthetized for the procedure, which alters LES pressure profiles and puts patients at heightened risk for aspiration pneumonia and GER. Additionally, variables including body position (244), obesity (340), and brachycephalic conformation (341) can alter the anatomy and mechanics of the EGJ junction. Finally, a logistical hurdle may be providing adequate training to personnel to ensure that EndoFLIP is conducted consistently and interpreted properly.

#### Future Directions in Veterinary Medicine

If the safety and feasibility of EndoFLIP is proven in dogs, the technology may be useful to characterize EGJ outflow obstruction, esophageal achalasia, or GERD disorders in dogs. It is also possible that EndoFLIP could document hiatal herniation in brachycephalic breeds with a higher intra-balloon pressure, a different anesthetic protocol, or the newer EndoFLIP 2.0 module. EndoFLIP could also be used in the operating room to assess GEJ distensibility and predict patient outcomes following fundoplication. EndoFLIP could potentially be used to evaluate dogs with cricopharyngeal disease, but first requires further study of the UES in humans. Furthermore, a study in healthy dogs showed greater variability in UES measurements and significant effects of body position (55). Other potential confounding variables such as patient size and body condition may also significantly affect measurements.

## Models for Translational Research and Medicine

A variety of animal models (rodent, canine, feline, opossum, porcine, and non-human primate) have been utilized to study human esophageal disorders, particularly GERD and the progression to Barrett's esophagus and esophageal adenocarcinoma (342, 343). While there are many advantages to using animal models, there are limitations as well. Rodent models are widely available, ideal for laboratory maintenance, and suitable for genetic modification, but are different to humans in various ways. Rodents have keratinized esophageal epithelium, lack submucosal glands and a squamocolumnar EGJ, and do not experience spontaneous reflux (343, 344). Furthermore, their esophageal musculature differs to that in humans and other animal species. Both the rodent and canine esophagus (35) consist of primarily striated muscle whereas cats (345), opossums (346), pigs (347), and non-human primates (348) have both striated and smooth muscle, as found in humans (349). Additionally, rodents and most animal species except for non-human primates, rarely develop Barrett's esophagus secondary to GER (350). Thus, surgical procedures (351–356) or intraesophageal infusions (357, 358) are required to induce reflux and esophageal injury, which raises concerns and questions regarding the ethics and utility of these representative models.

Nevertheless, due to the homology at the EGJ and shared esophageal physiology between humans and canines, there is opportunity for translational research. Many different canine models of GER already exist that have helped in understanding the pathogenesis of Barrett's esophagus in humans (302). Most are uncontrolled, artificial reflux models with surgically configured paraesophageal hiatal hernias, esophago-intestinal anastomoses, or biliary diversions that induce reflux (308, 343, 359). There are sporadic cases of Barrett's esophagus and adenocarcinoma in dogs (360, 361), but the process and progression may take longer than the median lifespan of a dog (362). It is also plausible that Barrett's esophagus is underrecognized because endoscopic esophageal biopsies are rarely obtained in dogs. Although Barrett's esophagus is infrequent in dogs, spontaneous canine models of GER in brachycephalics with hiatal herniation do occur. Many of these dogs also have BOAS making them optimal models of aerodigestive disease to study the effects of sleep apnea syndrome (363), evaluate the relationships of intrathoracic pressure alterations and reflux (364), and interrogate the response to corrective airway surgery (168, 365, 366). The role of acid and airway reflux in aspiration pneumonia (367), laryngeal dysfunction or spasm (368, 369), chronic cough and bronchoconstrictive airway disease (370) could also be studied in dogs and related back to in humans (371).

Canine models of GER also enable pilot testing of novel diagnostic and therapeutic approaches. Procedures such as CT (341), nuclear scintigraphy (289), or acoustic interrogation devices have been used to detect GER in dogs (372). Nuclear scintigraphy can also be used to detect silent, post-prandial, and extraesophageal reflux (289). Measurement of biomarkers like gastric pepsin in saliva (373) or bile acids in airway samples could confirm extraesophageal reflux and microaspiration (370). However, pepsin may not be a useful biomarker in dogs because concentrations are very low in canine gastric fluid. Furthermore, pepsin has not been detected in oropharyngeal swabs from dogs with a known history of vomiting or regurgitation (374). Bioacoustic recordings of respiratory sounds can be performed and synchronized with reflux testing to establish cough-reflux associations in aerodigestive patients (375). In addition to testing innovative diagnostic methods in dogs, novel treatments such as baclofen to reduce tLESr (376), injectable bulking agents for the LES (377), electrical microstimulation of the LES (378), extracellular matrix hydrogel to mitigate Barrett's esophagus (379), tissue engineering with autologous cells and/or bioscaffolds to prevent esophageal strictures (380) and repair esophageal defects (381–384), and surgical procedures, such as endoscopic fundoplication (385, 386), have already been trialed in canine models and are paving the way for usage in human patients.

Apart from GER models, there are spontaneous and induced canine models for cricopharyngeus muscle dysfunction and esophageal achalasia as well. Canine breeds such as the golden retrievers, miniature dachshunds, Maltese, and spaniels are highly predisposed to cricopharyngeus muscle dysfunction (92–94). Genomic analysis in affected breeds could elucidate hereditary mutations that develop genetic models of disease. For example, golden retrievers, Rottweilers, German shorthaired pointers, Welsh corgis, Cavalier King Charles spaniels, Cockers spaniels, Tibetan terriers, and Labrador retrievers are genetically predisposed to the X-linked recessive disorder, muscular dystrophy, due to a heritable mutation that depletes muscular dystrophin (Table 1) (387). Studying these canine models has helped isolate the culprit mutation and implement gene editing technology to cure the disease (388). Similar genomic analyses could be fruitful in patients with esophageal achalasia and secondary megaesophagus. Further study in dogs could also uncover environmental factors associated with esophageal achalasia and/or secondary megaesophagus, such as toxins (aflatoxin) (389) or dietary ingredients (390). Esophageal achalasia can even be experimentally induced in canines by injecting a surfactant, benzyldimethyltetradecylammonium chloride into the LES. Novel interventions or procedures for achalasia such as retrievable self-expanding cardia stents or esoFLIP (endoFLIP with balloon dilation) for esophageal achalasia (391) or cricopharyngeus per oral endoscopic myectomy (c-POEM) for cricopharyngeus achalasia can then be tested (392).

Diagnostics such as swallowing fluoroscopy, HRM, pH/impedance, and endoFLIP will be valuable in accurately assessing the therapeutic effects of such interventions. Incorporating these diagnostic tools into translational research and veterinary practice will require interdisciplinary collaboration between veterinary clinicians, human health care professionals, researchers, and biomedical device companies.

## Conclusion

Difficulty swallowing is a highly prevalent symptom that significantly affects quality of life and shortens life expectancy in both humans and dogs. Assessment of oropharyngeal and esophageal function is critical to appropriately diagnose and manage swallowing disorders. Although not discussed in detail in this manuscript, careful consideration of delayed gastric emptying in precipitating GER is important. Due to the shared features in pharyngeal and esophageal anatomy, physiology, and pathology between humans and canines, diagnostic tests such as swallowing fluoroscopy, FEES, HRM, pH/impedance, and endoFLIP can be utilized in both species. However, there are notable challenges and limitations to performing and interpreting these tests in dogs. Although further research of these modalities in canines is necessary, there is significant potential for translational research and clinical application in veterinary and human medicine.

## Author Contributions

TU primarily authored the manuscript and completed multiple revisions of the draft. SM helped with the outline of the manuscript, assisted with revisions of the manuscript, and provided several figures. PB edited the manuscript, provided guidance on the outline of the manuscript, and provided several figures. JC and JP edited the manuscript and provided several figures. All authors reviewed and approved the final draft of the manuscript.

## Funding

The authors received Open Access Funds from UC Davis to cover a portion of publication costs.

## Conflict of Interest

The authors declare that the research was conducted in the absence of any commercial or financial relationships that could be construed as a potential conflict of interest.

## Publisher's Note

All claims expressed in this article are solely those of the authors and do not necessarily represent those of their affiliated organizations, or those of the publisher, the editors and the reviewers. Any product that may be evaluated in this article, or claim that may be made by its manufacturer, is not guaranteed or endorsed by the publisher.
