# Supplementary material for: A Comparative Assessment of the Diagnosis of Swallowing Impairment and Gastroesophageal Reflux in Canines and Humans
Source: Front Vet Sci. 2022 Jun 9;9:889331. doi: 10.3389/fvets.2022.889331 (PMC9228035; doi:10.3389/fvets.2022.889331)
Supplement: Supplementary file 1 [file Table_1.DOCX]

Supplementary Material

**Supplementary Figure 1:** The Dog-SAT (Swallowing Assessment Tool) is a questionnaire that has been developed and is currently being validated to assess the signs and severity of swallowing impairment, reflux, and regurgitation in canine patients.

***Please circle the severity of the scenarios below: 0 = No problem; 4 = Severe problem***

Has the swallowing problem caused your dog to lose weight? 0 1 2 3 4

Does your dog have a problem swallowing water? 0 1 2 3 4

Does your dog have a problem swallowing food? 0 1 2 3 4

The swallowing problem occurs with canned food 0 1 2 3 4

The swallowing problem occurs with dry kibble 0 1 2 3 4

Does the swallowing appear painful? (vocalizing, panting) 0 1 2 3 4

Does your dog cough when it swallows? 0 1 2 3 4

Does your dog appear to choke or gag when it swallows? 0 1 2 3 4

Does your dog repeatedly swallow a single mouthful of food? 0 1 2 3 4

Has your dog’s bark changed in pitch or sound? 0 1 2 3 4

Does your dog have excessive drooling? 0 1 2 3 4

Does food or water come out of your dog’s nose? 0 1 2 3 4

Does food fall out of your dog’s mouth when it swallows? 0 1 2 3 4

Does your dog regurgitate after eating? 0 1 2 3 4

Does your dog regurgitate during increased activity/excitement? 0 1 2 3 4

Does your dog smack its lips? 0 1 2 3 4

Does your dog seem weak or have less ability to exercise? 0 1 2 3 4

**Created by Dr. S.L. Marks UC Davis School of Veterinary Medicine (06/2012)**
